# Compact homogeneous Leviflat CR-manifolds

**DOI:** 10.1007/s40627-021-00083-y

**Published:** 2021-07-15

**Authors:** A. R. Al-Abdallah, B. Gilligan

**Affiliations:** Department of Mathematics and Statistics, University of Regina, Regina, Canada

**Keywords:** Homogeneous CR-manifolds, Leviflat, Levi-foliation, Dense leaves, 32V99, 32M05

## Abstract

We consider compact Leviflat homogeneous Cauchy–Riemann (CR) manifolds. In this setting, the Levi-foliation exists and we show that all its leaves are homogeneous and biholomorphic. We analyze separately the structure of orbits in complex projective spaces and parallelizable homogeneous CR-manifolds in our context and then combine the projective and parallelizable cases. In codimensions one and two, we also give a classification.

## Introduction

Foliations arise in various ways. Locally, their geometry is clear and it is their global behavior that is of interest, particularly, since it is possible to have leaves in a foliation that are not even close to being homeomorphic. One spectacular manifestation of this phenomenon occurs in the now classic Reeb foliation of $S^{3}$ that has one compact leaf while all other leaves are non-compact and accumulate to the compact one.

In this paper, we study the geometry of compact homogeneous Leviflat CR-manifolds. These have the form $\varSigma = G/H$, where *G* is a Lie group acting on $\varSigma$ by CR-automorphisms. Because the CR-structure in the homogeneous setting is analytic, it turns out that there is a foliation of the CR-manifold whose leaves have tangent bundles corresponding to the distribution given by the zero spaces of the Levi form and we call this the Levi-foliation. We prove in Sect. 3.1 that the leaves of this Levi-foliation on the homogeneous CR-manifold are homogeneous themselves under the action of a complex Lie subgroup of *G*. This setting is very special, since this implies that all the leaves are biholomorphic to one another.

We first consider the parallelizable setting in the fourth section. In codimension less than or equal to two, the radical orbits are closed. The quotient by the radical orbits is a compact homogeneous space of a maximal complex semisimple factor of the Lie group *G*, again with discrete isotropy. It is known that every semisimple complex Lie group contains uniform, discrete subgroups and the structure of the corresponding homogeneous spaces is generally difficult to analyze. However, because the base of the radical fibration is compact and the interesting part of the geometry takes place in the radical orbits, we avoid having to deal with such complexities. Settings where the leaves are dense are the most interesting in our opinion and we give a classification of these, as well as the easier case where the leaves are not dense. The radical orbits are towers of Abelian complex Lie groups and the geometry of such towers is understood. There are also examples given to illustrate the theory in a concrete fashion.

Projective orbits are studied in section five. By a result of Chevalley, the orbits of the commutator subgroup of the complex hull $\widehat{G}$ of *G* are closed, since that group is acting as an algebraic group in this setting. Using methods from the theory of algebraic groups which are now at hand we show that the radical of $\widehat{G}$ is central and, as a consequence, that the leaves are always flag manifolds. In low codimension, there is not much room left for transversal directions and the classification of the surfaces that occur as the corresponding leaf-spaces is well known, see [39] and [22]. This yields the classification in the projective setting.

It is well known that every homogeneous CR-manifold admits a homogeneous fibration, called the CR-normalizer fibration, whose fiber is parallelizable and whose base is an orbit in some projective space [23, 35], etc. In the sixth section, we use this fibration in order to combine the previous results to get the classification in the general setting. In the final section, we show that the spaces under consideration always admit globalizations.

### Remark 1.1

The main results in this paper were in the first author’s Ph.D. thesis [2].

## Preliminaries

### CR-manifolds

We shall begin by collecting some basic facts about CR-manifolds. For the general theory and more details, we refer the reader to [3, 6, 8, 15, 17, 19, 26, 32, 33, 36].

A *CR-manifold* of type (*m*, *l*) is a pair $(\varSigma ,\mathcal {H})$ consisting of a smooth manifold $\varSigma$ of dimension *m* and a complex subbundle $\mathcal {H}$, called a *CR-structure*, of the complexified tangent bundle $T^{\mathbb {C}}\varSigma$ of complex rank *l* such that the following conditions hold:

1. $\mathcal {H}\cap {\bar{\mathcal {H}}}= (0)$, i.e., the zero section.
2. $\mathcal {H}$ is involutive, i.e., the Lie-product $[\zeta , \xi ]$ is a smooth section whenever $\zeta$ and $\xi$ are smooth sections of $\mathcal {H}$.

One observes that the CR-structure $\mathcal {H}$ satisfies the inequality $0\le l\le m/2$. In case $l=0$, we call $\mathcal {H}$ a *totally real* structure. We also remark form this definition the following.

1. If $\zeta _{p},\xi _{p}\in \mathcal {H}_{p}$ and $\mathfrak {R}\zeta _{p}=\mathfrak {R}\xi _{p}$, then $\xi _{p}=\zeta _{p}$. Thus, we can define an almost complex structure on the 2*l*-dimensional (real) subbundle $\mathfrak {R}\mathcal {H}=\mathcal {H}+\bar{ \mathcal {H}}$ by the following: If $\xi =X_{1}+iX_{2}\in \mathcal {H}$ then
  $$\begin{aligned} \mathcal {J}\; : \; \mathfrak {R}\mathcal {H}\;&\rightarrow \; \mathfrak {R}\mathcal {H}, \\ X_{1}\;&\mapsto \; X_{2}. \end{aligned}$$
2. The CR-structure $\mathcal {H}$ being involutive does not imply necessarily that $\mathfrak {R}\mathcal {H}$ is involutive. Nevertheless, for all $X,Y\in \mathfrak {R}\mathcal {H}$, one has $[X,Y]+[\mathcal {J}X,\mathcal {J}Y]\in \mathfrak {R}\mathcal {H}$ and the *Nijenhuis tensor*
  $$\begin{aligned} N(X,Y):=\mathcal {J}([X,Y]+[\mathcal {J}X,\mathcal {J}Y])-[X,\mathcal {J}Y]. -[\mathcal {J}X,Y]=0. \end{aligned}$$

One thus can redefine a CR-manifold to be a triple $(\varSigma ,\mathcal {R},\mathcal {J})$, where $\mathcal {R}$ is a real subbundle of $T\varSigma$ of rank 2*l* with an almost complex structure tensor $\mathcal {J}$ so that the pair $(\mathcal {R},\mathcal {J})$ has an everywhere vanishing Nijenhuis tensor.

A smooth embedding $\tau$ of a CR-manifold $(\varSigma , \mathcal {H})$ of type (*m*, *l*) into a complex manifold *X* is called a *CR-embedding* if $\tau (\varSigma )$ is a CR-manifold with a CR-structure $\mathcal {H}\tau (\varSigma ):= T^\mathbb {C}\tau (\varSigma )\cap T^{1,0}X$. In this case, we say that $\varSigma$ is a *CR-submanifold* of *X*. If $l=m-\dim _{\mathbb {C}} X$, then we say that $\varSigma$ is a *generic* CR-submanifold of *X* and the latter is a *complexification* of the CR-manifold $\varSigma$. We remark that, if $\varSigma$ has codimension *d* in its complexification *X*, i.e., $m = 2\dim _{\mathbb {C}}X - d$, then,

$$\begin{aligned} d = \dim _{\mathbb {C}}X-l = m-2l. \end{aligned}$$

Note that the integer *d* can be found without using $\dim _{\mathbb {C}}X$ explicitly in the calculation. Hence, we define the *codimension* of $\varSigma$ to be

$$\begin{aligned} \mathrm {codim}\; \varSigma := m\; -\; 2l. \end{aligned}$$

A smooth map *f* between two CR-manifolds $(\varSigma _{1},\mathcal {R}_{1}, \mathcal {J}_{1})$ and $(\varSigma _{2},\mathcal {R}_{2}, \mathcal {J}_{2})$ is called a *CR-map* if $f_{*}(\mathcal {R}_{1}|_{x})= \mathcal {R}_{2}|_{f(x)}$ and $\mathcal {J}_{2}\circ f_{*}= f_{*}\circ \mathcal {J}_{1}$. Analogously, a smooth fiber bundle $\varSigma _{1}\rightarrow \varSigma _{2}$ is called a *CR-bundle* if the bundle map is a CR-map. We now prove the following simple but crucial lemma.

#### Lemma 2.1

(The Codimension Lemma) *Let* $(E, {\mathcal {R}}{E})$ *and* $(B,{\mathcal {R}}{B})$ *be two CR-manifolds. Suppose that there exists a CR-fibration* $\pi \;:\; E\; \longrightarrow \; B$. *Then*, *the fiber* *F* *is a CR-manifold with a CR-structure* $\mathcal {R}F\cong \ker \pi _{*}|_{\mathcal {R}E}$. *Moreover*,

- $$\dim _{\mathbb {R}} E=\dim _{\mathbb {R}}F+\dim _{\mathbb {R}}B$$.
- $$\mathrm {codim }\; E=\mathrm {codim }\; F+\mathrm {codim }\; B$$.

#### Proof

Let $p\in E$ and $q:=\pi (p)$ and consider the differential map

$$\begin{aligned} \pi _{*}|_{T_{p}E}: T_{p}E\longrightarrow T_{q}B. \end{aligned}$$

Since $\pi ^{-1}(q)\cong F$ and $\pi$ is submersion onto, then $T_{p}F=\ker \pi _{*}|_{T_{p}E}$. Moreover, since $\pi _{*}(\mathcal {R}_{p}E)=\mathcal {R}_{q}B$ as $\pi$ is a CR-map, one has the following surjective linear map

$$\begin{aligned} \pi _{*}|_{\mathcal {R}_{p}E}:\mathcal {R}_{p}E\longrightarrow \mathcal {R}_{q}B \end{aligned}$$

with

$$\begin{aligned} \mathcal {R}_{p}F\; :=\; \ker \pi _{*}|_{\mathcal {R}_{p}E}. \end{aligned}$$

Since $\dim \mathcal {R}_{p}E$ (resp. $\dim \mathcal {R}_{q}B$) is independent of the choice of $p\in E$ (resp. $q\in B$), and $\dim \mathcal {R}_{p}E=\dim \mathcal {R}_{p}F+\dim \mathcal {R}_{q}B$, we deduce that $\dim \mathcal {R}_{p}F$ is constant for all $p\in \varSigma$. Thus, one can define the vector subbundle $\mathcal {R}F:=\bigcup \nolimits _{p\in \varSigma } \mathcal {R}_{p}F$ of $\mathcal {R}E$ and provide it with an almost complex structure by restricting the almost complex structure of $\mathcal {R}E$. Clearly, this almost complex structure on $\mathcal {R}F$ satisfies condition $(2^{\prime })$ above. Thus, $(F, \mathcal {R}F)$ is a CR-manifold, and the first part of the lemma is proved.

Now, we prove the second part of the lemma. For part (a), let $U\subset B$ be a local trivialization of the bundle, i.e., $\pi ^{-1}(U)\cong F\times U$. Hence,

$$\begin{aligned} \dim _{\mathbb {R}}E=\dim _{\mathbb {R}}\pi ^{-1}(U)=\dim _{\mathbb {R}}U+\dim _{\mathbb {R}}F =\dim _{\mathbb {R}}B+\dim _{\mathbb {R}}F. \end{aligned}$$

For part (b), one has the following two equations:

$$\begin{aligned} \dim _{\mathbb {R}}T_{p}E= & {} \dim _{\mathbb {R}}T_{p}F\;+\; \dim _{\mathbb {R}}T_{q}B,\\ \dim _{\mathbb {R}}\mathcal {R}_{p}E= & {} \dim _{\mathbb {R}}\mathcal {R}_{p}F\; +\; \dim _{\mathbb {R}}\mathcal {R}_{q}B. \end{aligned}$$

Therefore,

$$\begin{aligned} \mathrm {codim}\; E=\mathrm {codim}\; F+\mathrm {codim}\; B. \end{aligned}$$

$$\square $$

A vector field $\xi \in \varGamma (\varSigma , T\varSigma )$ is called a *CR-vector field* if the local one-parameter group of transformations induced by $\xi$ consists of CR-transformations.

A CR-manifold $(\varSigma , \mathcal {H})$ is called *analytic* if $\varSigma$ is a real-analytic manifold and $\mathcal {H}$ is locally generated by real-analytic local sections in $T^{\mathbb {C}}\varSigma$. In other words, $\mathcal {H}$ is locally generated by complex-valued vector fields whose coefficient functions are real-analytic. Such manifolds satisfy strong properties (see [3]); Every analytic CR-manifold has a complexification. Moreover, if $f:\varSigma _{1}\rightarrow \varSigma _{2}$ is an analytic CR-map between two analytic CR-manifolds, then there exist complex *tubes* *U* and *V* containing $\varSigma _{1}$ and $\varSigma _{2}$ , respectively, and a holomorphic map $\widehat{f}:U\rightarrow V$ with $\widehat{f}|_{\varSigma }=f$. Similarly, if $\xi \in T\varSigma$ is an analytic vector field then the local CR-transformations in $\varSigma$ induced by $\xi$ extend to holomorphic transformations on a tube *U* around $\varSigma$. Those holomorphic transformations induce a holomorphic vector field $\eta$ on *U* with $\xi (p)=\mathfrak {R}\eta (p)$ for all $p\in \varSigma$.

On the other hand, if $(\varSigma ,\mathcal {H})$ is a CR-manifold then, as noted above, the distribution $\mathfrak {R}\mathcal {H}$ is not necessary integrable (i.e., not involutive). To measure the degree to which this distribution fails to be involutive, we introduce the so-called *Levi form* to be the vector valued 2-form, for all $x\in \varSigma$

$$\begin{aligned} L: \mathcal {H}\times \mathcal {H}\;&\rightarrow \; T^{\mathbb {C}}\varSigma /\mathcal {H}+ \bar{\mathcal {H}}\\ L(\zeta _{x},\xi _{x})\;&=\; \pi _{x}\big ([\zeta ,\bar{\xi }]_{x}\big ), \end{aligned}$$

where $\pi$ is the canonical projection of $T^{\mathbb {C}}\varSigma$ onto $T^{\mathbb {C}}\varSigma /\mathcal {H}+\bar{\mathcal {H}}$. Clearly, if *L* degenerates everywhere, then $\mathfrak {R}\mathcal {H}=\mathcal {H}+ \bar{\mathcal {H}}$ is involutive. In this case, $(\varSigma ,\mathcal {H})$ is said to be *Leviflat*. It follows by Frobenius Theorem that $\varSigma$ is foliated by complex leaves. If in addition this manifold is analytic, then the following theorem, due to M. Freeman (see Theorem 5.1 and its corollary in [17]) and C. Rea (see [33]), gives a more explicit local picture of this foliation. We state it in the following way.

#### Theorem 2.1

(Freeman 1974) *Let* $\varSigma$ *be a real-analytic CR-manifold of codimension* *k*, *and let* *X* *be a complexification of* $\varSigma$. *Then* $\varSigma$ *is Leviflat if and only if for each* $x\in \varSigma$ *there exists a local model* $(U,\varphi )$ *consisting of an open neighborhood* $U\subset X$ *of* *x* *and a biholomorphic map* $\varphi :U\rightarrow \varDelta _{k}\times \varDelta _{n-k}$ *where* $\varDelta _{d}$ *denotes a polydisk of dimension* *d* *in* $\mathbb {C}^{d}$ *containing the origin. Furthermore*,

$$\begin{aligned} \varphi (\varSigma \cap U)= (\varDelta _{k}\cap \mathbb {R}^{k})\times \varDelta _{n-k}. \end{aligned}$$

As a consequence, if $\varSigma$ a Leviflat analytic CR-manifold of codimension *k* and if *X* is a complexification of complex dimension *n*, then $\varSigma$ is foliated by complex manifolds $\mathscr {F}:=\{\mathcal {L}_{\alpha }\}$ where each *complex leaf* $\mathcal {L}_{\alpha }\in \mathscr {F}$ is locally biholomorphic to $\varDelta _{n-k}$. The foliation $\mathscr {F}$ is called the *Levi-foliation* on $\varSigma$.

### Homogeneous CR-manifolds

The main references for this section are [6, 19, 35], where the reader can also find more details.

A CR-manifold $(\varSigma ,\mathcal {H})$ is called a *homogeneous CR-manifold* if there exists a Lie group *G* acting transitively on $\varSigma$ as a group of CR-automorphisms. It is proved in [35, Zusatz zu Satz 2] that $\mathcal {H}$ is locally generated by analytic sections in $T^\mathbb {C} \varSigma$. Therefore, since smooth homogeneous manifolds are analytic, then every homogeneous CR-manifold has a complexification.

If we assume that the action of *G* on $\varSigma$ is *almost effective*, then we can identify the Lie algebra $\mathfrak {g}$ of the Lie group *G* with *CR-fundamental vector fields* in the Lie algebra of CR-vector fields on $\varSigma$. As a result, for every $\xi \in \mathfrak {g}$ there exists a tube $U_{\xi }$ containing $\varSigma$ in *X* such that $\xi$ extends to a holomorphic vector field $\eta$ on $U_{\xi }$ so that $\mathfrak {R}\eta =\xi$. Moreover, since ${\mathfrak {g}}$ is *finite dimensional*, we can redefine the complexification *X* of $\varSigma$ to be the intersection of all tubes of the corresponding CR-vector fields that form a finite basis of ${\mathfrak {g}}$. Consequently, every CR-vector field on $\varSigma$ extends holomorphically and *uniquely* on *X*, and hence we can define $\hat{\mathfrak {g}}$ to be the *complex Lie algebra* that consists of all those extended holomorphic vector fields. This being so, any complex Lie group $\widehat{G}$ with Lie algebra $\widehat{\mathfrak {g}}$ acts locally and holomorphically on *X*. In an ideal situation, this local action is globalizable.

To summarize, suppose that $\varSigma =G/H$ is a homogeneous CR-manifold where *H* is a closed subgroup of *G*, and let $\widehat{G}$ be a complex Lie group with Lie algebra $\widehat{\mathfrak {g}}$. If the complexification *X* of $\varSigma$ can be taken to be the homogeneous complex manifold $\widehat{G}/\widehat{H}$, where $\widehat{H}\cap G=H$, i.e., $\varSigma$ is the orbit of the subgroup *G* in *X*, then we say that $\varSigma$ is globalizable and *X* is its globalization.

A generalization of the normalizer fibration of complex homogeneous manifolds (see e.g., [39]) is the *CR-normalizer fibration* (an analog of the $\mathfrak {g}$-anticanonical fibration first introduced by Huckleberry and Oeljeklaus [23]. See also [6] for more details. This fibration plays a central role in our classification in the final section.

#### Theorem 2.2

(CR-normalizer fibration) *Let* $\varSigma =G/H$ *be a homogeneous CR-manifold*, *where* *G* *is a connected Lie group and* *H* *is a closed subgroup of* *G*. *Then there exists a closed subgroup* *J* *of* *G* *containing* *H*,  *such that base of the following fibration is CR-equivariantly embedded in some projective space* $\mathbb {P}_{k}$.

1 $$\begin{aligned} \varSigma = G/H \xrightarrow { F } \;G/J \; \hookrightarrow \mathbb {P}_{k}. \end{aligned}$$

*Moreover*, *J* *is contained in the normalizer of the connected component* $H^{0}$ *of* *H* *in* *G*. *Thus*, *the fiber* $F=J/H$ *is a parallelizable homogeneous CR-submanifold of* $\varSigma ,$ *i.e.*, $F= L/\varGamma$ *for a discrete subgroup* $\varGamma :=H/H^{0}$ *of the Lie group* $L:=J/H^{0}$.

Note that since the base *G*/*J* of the normalizer fibration is a projective space, then it always possesses a globalization $\widehat{G}/\widehat{J}$, where $\widehat{G}$ is a complex Lie subgroup of the $\mathrm {PSL}_{n+1}(\mathbb {C})$. On the other hand, we will see that the parallelizable fiber $F=L/\varGamma$ also possesses a globalization $\widehat{L}/\varGamma$ when its codimension is less than or equal two (see Sect. 4).

## Leviflat homogeneous CR-manifolds

### Homogeneity of the leaves

#### Lemma 3.1

*Let* $\mathcal {L}$ *be any leaf in the Levi-foliation* $\mathscr {F}$ *of a Leviflat homogeneous CR-manifold* $\varSigma =G/H$. *Then*, *for any* $g\in G,$ *either* $(g\cdot \mathcal {L})\cap \mathcal {L}=\varnothing$ *or* $g\cdot \mathcal {L}=\mathcal {L}$.

#### Proof

Suppose there exists $g\in G$ with $g\mathcal {L}\cap \mathcal {L}\not =\varnothing$. In order to show the set

$$\begin{aligned} Y:=\{y\in \mathcal {L};\ g\cdot y\in \mathcal {L}\} \end{aligned}$$

is the whole leaf $\mathcal {L}$, we show that it is both closed and open:

(Closed) Given a sequence $\{y_{n}\}\subset Y$ such that it converges to $y\in \mathcal {L}$. We need to show that $gy\in \mathcal {L}$ because then we observe that $y\in Y$. Indeed, let *W* be a ‘leaf-chart’ containing *y*, i.e., $\varphi (\mathcal {L}\cap W)=\varphi (W)\cap \mathbb {R}^{d}$. Thus, $\mathcal {L}\cap W=F^{-1}(0)$ where $F:=\pi \circ \varphi$ and $\pi :\mathbb {R}^{n}\rightarrow \mathbb {R}^{n-d}$ is the projection. Hence, $W\cap \mathcal {L}$ is a closed subset of $\mathcal {L}$, which hence implies that $gW\cap \mathcal {L}$ is also a closed subset of $\mathcal {L}$. Therefore, $gW\cap \mathcal {L}$ contains $gy_{n}\rightarrow gy$ for all $n\ge N$ for sufficiently large *N*, i.e., $gy\in \mathcal {L}$, as wanted.
(Open) Let *U* be an open neighborhood of a point $x\in Y$ in the leaf $\mathcal {L}$. Since *g* is a CR-automorphism, *g*(*U*) is a (local) open complex manifold in $\varSigma$. But $\mathcal {L}$ locally is the unique such manifold (see Theorem 2.1) and since $g(U)\cap \mathcal {L}\not =\varnothing$, it follows that $g(U) \subset \mathcal {L}$. So $U\subset Y$ and *Y* is open.

$$\square $$

#### Corollary 3.1

(Leaf-stabilizer) *Let* $\mathcal {L}$ *be the leaf of the Levi-foliation* $\mathscr {F}$ *through the base point* $x_{0}\in \varSigma$. *Then there exists a connected* (*not necessarily closed*) *Lie subgroup* $G_{\mathcal {L}}$ *of* *G*,  *called the* **leaf-stabilizer**, *such that* $\mathcal {L}=G_{\mathcal {L}}\cdot x_{0}$. *In particular*, *for all* $g\in G,$ *the group* $gG_{\mathcal {L}}g^{-1}$ *stabilizes the leaf* $g\mathcal {L}$ *through the point* $x_{1}:=g\cdot x_{0},$ *and hence* $\mathscr {F}=\{g\mathcal {L}\}_{g\in G}=\{gG_{\mathcal {L}}g^{-1}\cdot x_{0}\}_{g\in G}$.

As a consequence of Corollary 3.1, we have the following: since the restriction of the group *G*-action on $\varSigma$ to any leaf $\mathcal {L}\in \mathscr {F}$ is holomorphic, *all leaves of the Levi-foliation are biholomorphic*. In particular, if one leaf is compact (resp. dense) in $\varSigma$ then all leaves of the Levi-foliation are compact (resp. dense) in $\varSigma$.

#### Corollary 3.2

*Let* $\mathcal {L}\in \mathscr {F}$ *be the leaf through the base point* $x_{0}\in \varSigma =G/H$ *and let* $G_{\mathcal {L}}$ *be the leaf-stabilizer. Then the isotropy subgroup* *H* *stabilizes* $\mathcal {L}$. *Consequently*, *the connected component* $H^{\circ}$ *of* *H* *is contained in* $G_{\mathcal {L}},$ *and moreover* *H* *normalizes* $G_{\mathcal {L}}$. *In particular*, $G_{\mathcal {L}}H$ *is a Lie subgroup of* *G*.

#### Proof

Clearly, $x_{0}\in \mathcal {L}\cap h(\mathcal {L})$ for all $h\in H$. Then, by Lemma 3.1, $h(\mathcal {L})=\mathcal {L}$ for all $h\in H$. Hence, since the leaf-stabilizer $G_{\mathcal {L}}$ is connected, we have $H^{\circ}$ is a Lie subgroup of $G_{\mathcal {L}}$. Furthermore, the connected group $hG_{\mathcal {L}}h^{-1}$ (see Corollary 3.1) stabilizes the leaf $h\mathcal {L}=\mathcal {L}$. Thus $hG_{\mathcal {L}}h^{-1}=G_{\mathcal {L}}$ for all $h\in H$. $\square$

In particular, if the leaf $\mathcal {L}$ is compact (hence all leaves are compact) then the Lie subgroup $G_{\mathcal {L}}\cdot H$ is a closed subgroup of *G* and hence we have the following fiber bundle

$$\begin{aligned} \varSigma = G/H\longrightarrow G/G_{\mathcal {L}}H. \end{aligned}$$

We will discuss this fibration in more detail in the next section.

#### Corollary 3.3

*Let* *M* *be the connected complex Lie subgroup of* *G* *corresponding to the maximal complex Lie subalgebra* $\mathfrak {m}:=\mathfrak {g}\cap i\mathfrak {g}$ *of* $\mathfrak {g}$. *Then* $M\subset G_{\mathcal {L}}$.

#### Proof

The holomorphic orbit $M\cdot x_{0}$ is a complex submanifold of $\varSigma$. However, by Freeman’s Theorem 2.1, the leaf $\mathcal {L}$, locally, is the unique maximum complex submanifold of $\varSigma$ passing through the base point $x_{0}$. Thus, locally we have $M\cdot x_{0}\subset \mathcal {L}$. The desired result follows by repeating the local process along the *M*-orbit.$\square$

#### Proposition 3.1

(Dense Leaves) *Let* $\varSigma =G/H$ *be a Leviflat homogeneous CR-manifold and let* $\mathscr {F}$ *be its Levi-foliation. Suppose that one of the leaves of* $\mathscr {F}$ *is dense in* $\varSigma$. *Then all leaves of* $\mathscr {F}$ *are dense in* $\varSigma ,$ *and the leaf-stabilizer* $G_{\mathcal {L}}$ *is a normal Lie subgroup of* *G*.

#### Proof

Let $\mathcal {L}$ and $\mathcal {L}_{1}$ be two dense leaves, then we show that $\mathfrak {g}_{\mathcal {L}}=\mathfrak {g}_{\mathcal {L}_{1}}$ where $\mathfrak {g}_{\mathcal {L}}$ and $\mathfrak {g}_{\mathcal {L}_{1}}$ are the Lie algebras of $G_{\mathcal {L}}$ and $G_{\mathcal {L}_{1}}$. It suffices to show that in some local model

$$\begin{aligned} \pi :U:= \varDelta _{k}\times \varDelta _{n-k}\longrightarrow \varDelta _{k} \end{aligned}$$

every holomorphic vector field $\widehat{\xi }$ defined by $\xi \in \mathfrak {g}_{\mathcal {L}}$ is tangent to every $\pi$-fiber.

Let $\mathcal {L}$ locally accumulate to the natural $\pi$-fiber. In concrete terms, let $(z^{1},\ldots ,z^{n})$ be polydisk coordinates of *U* and for $\xi \in \mathfrak {g}_{\mathcal {L}}$ write

$$\begin{aligned} \widehat{\xi }\; =\; \sum _{j=1}^{n}\; h_{j}\dfrac{\partial }{\partial z_{j}} \end{aligned}$$

for some holomorphic functions $h_{j}:U\rightarrow \mathbb {C}$. Since $\widehat{\xi }$ is tangent to $\mathcal {L}$, there exists a sequence $(z_{m}^{1},\ldots ,z^{k}_{m})$ that converges to 0 in $\varDelta _{k}$ so that

$$\begin{aligned} h_{j}(z_{m}^{1},\ldots ,z^{k}_{m},\cdot )=0 \end{aligned}$$

for all *m*. Thus, the complex variety

$$\begin{aligned} V:= \big \{h_{1}=h_{2}=\cdots =h_{k}=0\big \} \end{aligned}$$

contains the sequence

$$\begin{aligned} V_{m}= \big \{h_{1}(z_{m}^{1},\ldots ,z^{k}_{m},\cdot )= \cdots =h_{k} (z_{m}^{1},\ldots ,z^{k}_{m},\cdot )=0\big \} \end{aligned}$$

of local subvarieties which accumulates to the natural $\pi$-fiber and consequently $h_{1},\ldots ,h_{k}$ vanish identically on *U*. This is equivalent to $\widehat{\xi }$ being tangent to every local leaf in *U* and the desired result follows by the identity principle. $\square$

In case $\varSigma =G/H$ possesses a globalization $X:=\widehat{G}/\widehat{H}$, we have the following generalization of the above discussion.

#### Lemma 3.2

*Suppose that* $X=\widehat{G}/\widehat{H}$ *is a globalization of the Leviflat homogeneous CR-manifold* $\varSigma =G/H$. *Define the* **complex stabilizer** *of the leaf* $\mathcal {L}$ *through the base point* $x_{0}$ *to be the connected complex Lie subgroup* $\widehat{G_{\mathcal {L}}}$ *of* $\widehat{G}$ *corresponding to the complex Lie subalgebra* $\widehat{\mathfrak {g}_{\mathcal {L}}}:=\mathfrak {g}_{\mathcal {L}}+i\mathfrak {g}_{\mathcal {L}}$ *of* $\widehat{\mathfrak {g}}$. *Then* $\widehat{G_{\mathcal {L}}}$ *has the following properties*:

- *The leaf* $\mathcal {L}$ *is the holomorphic orbit of* $\widehat{G_{\mathcal {L}}}$ *through the base point.*
- *The connected component* $\widehat{H}^{\circ}$ *of the isotropy* $\widehat{H}$ *is contained in* $\widehat{G_{\mathcal {L}}}$.

#### Proof

- Follows from the definition of $\widehat{G_{\mathcal {L}}}$.
- At the Lie algebra level and by Corollary 3.3, we have $\mathfrak {m}=\mathfrak {g}\cap i\mathfrak {g}\subset \mathfrak {g}_{\mathcal {L}}$. Now write $\widehat{\mathfrak {g}}$ and $\widehat{\mathfrak {g}_{\mathcal {L}}}$ as follows:
  $$\begin{aligned}&\widehat{\mathfrak {g}} \;\;=\;\; \mathfrak {g}/\mathfrak {m} \;\;\oplus \;\; i(\mathfrak {g}/\mathfrak {m}) \;\;\oplus \;\; \mathfrak {m},\\&\widehat{\mathfrak {g}_{\mathcal {L}}} \;\;=\;\; \mathfrak {g}_{\mathcal {L}}/\mathfrak {m}\; \oplus \; i(\mathfrak {g}_{\mathcal {L}}/\mathfrak {m})\;\oplus \;\;\mathfrak {m}. \end{aligned}$$
  Consequently, the complex codimension of the Lie algebra $\widehat{\mathfrak {g}_{\mathcal {L}}}$ in $\widehat{\mathfrak {g}}$ is equal to the real codimension of $\mathfrak {g}_{\mathcal {L}}$ in $\mathfrak {g}$ and we have the following equalities:
  $$\begin{aligned} \dim _{\mathbb {C}}\widehat{G}-\dim _{\mathbb {C}}\widehat{G_{\mathcal {L}}} \;\;=&\dim _{\mathbb {R}} G-\dim _{\mathbb {R}} {G_{\mathcal {L}}}\\ \;\;\overset{{\text {Cor}}. 3.2}{=}&\dim _{\mathbb {R}}\varSigma -\dim _{\mathbb {R}} \mathcal {L}\\ \;\;=\;\;&\dim _{\mathbb {C}}X-\dim _{\mathbb {C}}\mathcal {L}\\ \;\;=\;\;&\dim _{\mathbb {C}}\widehat{G}/\widehat{H}-\dim _{\mathbb {C}}\widehat{G_{\mathcal {L}}}/\widehat{G_{\mathcal {L}}} \cap \widehat{H}. \end{aligned}$$
  Therefore, $\dim _{\mathbb {C}}\widehat{H}=\dim _{\mathbb {C}}\left( \widehat{G_{\mathcal {L}}} \cap \widehat{H}\right)$. Which implies $\widehat{H}^{\circ} \subset \widehat{G_{\mathcal {L}}}$ as desired.

$$\square $$

#### Remark 3.1

One can define the complex stabilizer of the leaf $\mathcal {L}$ in the above lemma to be the (possibly not connected) complex Lie group

$$\begin{aligned} \widehat{J}:=\left\{ g\in \widehat{G}; \;\; g(\mathcal {L})\subset \mathcal {L} \right\} . \end{aligned}$$

Clearly, we have $\widehat{H}^{\circ} ,H\subset \widehat{J}$. We shall henceforth abuse notation and write $\widehat{G_{\mathcal {L}}}$ for $\widehat{J}$.

### Minimality condition

The globalization $\widehat{G}/\widehat{H}$ is not unique. For example, if $G/H=S^{1}$, then $\widehat{G}/\widehat{H}$ can be $\mathbb {C}^{*}$, a 1-dimensional complex torus, or $\mathbb {P}_{1}$ (e.g., see Theorem 5.3). Furthermore, in a way that can be easily determined, such phenomena can arise in more complicated examples. To remedy this we impose the minimality condition that $\widehat{H}=H\widehat{H}^{\circ}$. This can always be arranged by noticing that the quotient $\widehat{H}/(H\widehat{H}^{\circ} )$ is discrete and

$$\begin{aligned} \widehat{G}/(H\widehat{H}^{\circ} ) \longrightarrow \widehat{G}/\widehat{H} \end{aligned}$$

is a covering which is biholomorphic over $\varSigma$. One then replaces $\widehat{H}$ by $H\widehat{H}^{\circ}$.

Recall that it has been shown that $\widehat{H}^{\circ} \subset \widehat{G_{\mathcal {L}}}$ (see Lemma 3.2). Therefore, under the *minimality assumption* it follows that $\widehat{H}\subset \widehat{G_{\mathcal {L}}}$ where $\widehat{G_{\mathcal {L}}}$ is defined to be the (possibly not connected) stabilizer of $\mathcal {L}$ in $\widehat{G}$ (see Remark 3.1). The connected component of the identity of this complex Lie group is well defined. But since $\mathcal {L}$ is at first not known to be closed, in arguments where this stabilizer is needed we go to a covering and we simply defined the leaf-stabilizer to be $\widehat{H}\widehat{G_{\mathcal {L}}}^{\circ}$ or equivalently $H\widehat{G_{\mathcal {L}}}^{\circ}$.

### The role of compactness of leaves

Let $\mathscr {L}$ be a foliation on a smooth manifold *M*. Define the **leaf-space** $M/\mathscr {L}$ of $\mathscr {L}$ to be the set of equivalence classes, where two points are equivalent if and only if they lie in the same leaf of the foliation. This space may have a complicated topological structure, as it is not necessarily Hausdorff even if all leaves are compact (see e.g., [38]). However, Reeb showed in his thesis [34] that if all leaves are compact and of codimension one, then the leaf-space is Hausdorff. Nevertheless, we will see that when $\varSigma$ is a Leviflat homogeneous CR-manifold and all leaves of the Levi-foliation $\mathscr {F}$ are compact, then the leaf-space $\varSigma /\mathscr {F}$ is always a reasonable homogeneous CR-manifold.

Now, let $\varSigma =G/H$ be a Leviflat homogeneous CR-manifold of codimension *k*, and let $\mathscr {F}$ be the Levi-foliation on $\varSigma$. Suppose the leaf $\mathcal {L}=G_{\mathcal {L}}\cdot x_{0}$ through the base point in $\varSigma$ is compact. Then, $G_{\mathcal {L}}H$ is a closed subgroup of *G*. Therefore, we have the (homogeneous) *leaf-reduction*

$$\begin{aligned} \varSigma \; =\; G/H\; \longrightarrow \; G/G_{\mathcal {L}} H\;=\;\varSigma /\mathscr {F}. \end{aligned}$$

The base is a *k*-dimensional homogeneous manifold. Locally, the leaf-reduction is equivalent to a projection (see Freeman’s Theorem 2.1)

$$\begin{aligned} (\varDelta _{k}\cap \mathbb {R}^{k})\times \varDelta _{n-k}\; \longrightarrow \; (\varDelta _{k}\cap \mathbb {R}^{k}). \end{aligned}$$

Since any two local models are holomorphically equivalent, it follows that $\varSigma /\mathscr {F}$ has the structure of a *k*-dimensional CR-manifold so that $\varSigma \rightarrow \varSigma /\mathscr {F}$ is a CR-bundle. Since the CR-automorphisms of $\varSigma$ act holomorphically on the local models, *G* acts as a group of CR-automorphisms on the base. Summarizing, we have the following situation in the case of compact leaves.

#### Proposition 3.2

(Compact leaves) *Let* $\varSigma =G/H$ *be a Leviflat homogeneous CR-manifold of codimension* *k*. *If some leaf* $\mathcal {L}$ *is compact*, *then every leaf is compact*, *and the leaf-space* $\varSigma /\mathscr {F}$ *is Hausdorff in the quotient topology. This leaf-space has a canonically defined* *k*-*dimensional * (*totally real*) *homogeneous CR-manifold structure. The reduction map* $\varSigma \rightarrow \varSigma /\mathscr {F}$ *is a CR-bundle and* *G*-*homogeneous, being realized as the homogeneous fibration*

$$\begin{aligned} \varSigma \;=\;G/H\;\longrightarrow \; G/G_{\mathcal {L}}H\;=\; \varSigma /\mathscr {F}\; =:\; \varSigma _{\text {red}} \end{aligned}$$

*with* *G* *acting as a group of CR-automorphisms on the base.*

In particular, if $\varSigma$ is compact and has codimension *k* then $\varSigma _{\text {red}}$ is also compact and has dimension *k*. For example, if $k=1$ then $\varSigma _{\text {red}}=S^{1}$. Or if $k=2$, then $\varSigma _{\text {red}}$ is $S^{2}$, $\mathbb {RP}_{2}$, $S^{1}\times S^{1}$, or the Klein bottle (see [28]).

In passing, we note that if $\varSigma$ is not compact, then $\varSigma _{\text {red}}$ need not be compact, as well. In codimension one we could have $\varSigma _{\text {red}}=\mathbb {R}$, and in codimension two $\varSigma _{\text {red}}= \mathbb {R} ^{2}$, $S^{1}\times \mathbb {R}$, or the Möbius strip. This completes the list of 2-dimensional homogeneous manifolds under the action of a Lie group in [28].

## Parallelizable CR-manifolds

We discuss in this section compact parallelizable homogeneous CR-manifolds. They have the form $G/\varGamma$, where *G* is a simply-connected real Lie group and $\varGamma$ is a discrete subgroup of *G*. Under some mild restrictions we show that the radical orbits are closed. In codimensions one and two this is sufficient in order to show that a certain tower of Cousin groups exists and contains the structure. We also prove, among other results, the existence of the globalization $\widehat{G}/\varGamma$ under the assumption that the CR-manifold has codimension less than or equal two. This section ends with a notable example.

### Cousin groups

Let $\widehat{\mathfrak {g}}:= \mathfrak {g}+i\mathfrak {g}$ be the complexification Lie algebra $\mathfrak {g}$ of *G*, and consider the maximal complex ideal $\mathfrak {m}:=\mathfrak {g}\cap i\mathfrak {g}$ of $\mathfrak {g}$. Since $\dim _{\mathbb {R}} \widehat{\mathfrak {g}} = 2 \dim _{\mathbb {R}} \mathfrak {g} - \dim _{\mathbb {R}} \mathfrak {m}$, one observes that $\dim _{\mathbb {R}} \widehat{\mathfrak {g}} - \dim _{\mathbb {R}} \mathfrak {g} \;=\; \dim _{\mathbb {R}} \mathfrak {g} - \dim _{\mathbb {R}} \mathfrak {m}$, i.e., the real codimension of $\mathfrak {m}$ in $\mathfrak {g}$ is equal to the real codimension of $\mathfrak {g}$ in $\widehat{\mathfrak {g}}$. Therefore, if *M* is the connected normal subgroup of *G* corresponding to $\mathfrak {m}$, then $\varSigma =G/\varGamma$ is a Leviflat generic homogeneous CR-manifold, and the leaves of the Levi-foliation are the *M*-orbits. Note that *M*-orbits may or may not be closed and the discussion so far is very general.

By definition, a connected complex Lie group that has no non-constant holomorphic functions is called a Cousin group. Since the adjoint representation of the group maps into some $\mathrm {GL}(n, \mathbb {C})$ and the latter is holomorphically separable, it follows that this representation is trivial. But the kernel of the adjoint representation is central and thus *every Cousin group is Abelian*. Now a connected Abelian complex Lie group *G* is the quotient of some vector space $\mathbb {C}^{n}$ by a discrete subgroup $\varGamma$ which has rank $n+k$ with $1 \le k \le n$ in our setting. Hence its topological structure is known, namely it is isomorphic (as real Lie groups) to a product $V_{\varGamma }/\varGamma \times \mathbb {R}^{n-k}$, where $V_{\varGamma }$ denotes the real space of $\varGamma$ in $\mathbb {C}^{n}$. Thus $K := V_{\varGamma }/\varGamma$ is the maximal compact subgroup of *G* and is isomorphic to $(S^{1})^{n+k}$.

It is interesting to note that *G* can be written as a quotient of $(\mathbb {C}^{*})^{n}$ by a discrete subgroup $\varLambda$ of rank $n-k$ and that the Cousin group $G = \mathbb {C}^{n}/\varGamma$ fibers (in many ways) as a $(\mathbb {C}^{*})^{n-k}$-bundle over a complex torus *T*, e.g., see Abe–Kopfermann [1]. Let *Y* be the corresponding $(S^{1})^{n-k}$-subbundle over *T* for any such choice. From the point of view of complex geometry it is essential to note that the *M*-orbits in *Y* are dense and form an infinite-to-one covering of the base torus *T*.

In the next section, we continue our investigation of Levi-foliations of compact CR-manifolds of the form $\varSigma :=G/\varGamma$ where $\varGamma$ is a discrete subgroup of the real Lie group *G*. Perhaps, surprisingly, the basic building blocks that can occur in this setting are compact homogeneous complex manifolds and fiber bundles involving powers of $S^{1}$ lying inside corresponding powers of $\mathbb {C}^{*}$-bundles in *X*, as we noted above. We will outline how this happens, even in the setting where the leaves are dense—so no reasonable (i.e., Hausdorff) leaf-space exists. This gives a rather explicit description of the structure even in this setting. When the leaves of the Levi-foliation are compact then the *leaf-space* of the Levi-foliation is just the base of the leaf-reduction fibration $G/\varGamma \xrightarrow { M/M\cap \varGamma } G/M\varGamma$ while the leaves are nothing but the fibers $M/M\cap \varGamma$, where *M* is the connected complex Lie subgroup correspondent to maximal complex ideal $\mathfrak {m}=\mathfrak {g}\cap i\mathfrak {g}$.

### Building blocks in the setting of dense leaves

We first recall the fact that any connected and simply-connected complex solvable Lie group admits a faithful representation. Moreover, it is biholomorphic (as manifolds) to some $\mathbb {C}^{n}$ and its connected Lie subgroups are closed and simply-connected, see [13]. The following theorem (a special case of [35, Satz 1.4.2.1]) ensures the existence of the globalization $\widehat{G}/\varGamma$ of $G/\varGamma$ (see also [19, Theorem 2.7]).

#### Theorem 4.1

*Let* *G* *be a connected and simply-connected Lie group with Lie algebra* $\mathfrak {g}$. *Suppose that* $\varSigma :=G/\varGamma$ *is a compact parallelizable homogeneous manifold of codimension* (*i.e., the real codimension of the maximal complex ideal* $\mathfrak {m}:=\mathfrak {g}\cap i\mathfrak {g}$ *in* $\mathfrak {g}$) *less than or equal to* 2. *Then* *G* *is a closed subgroup of the connected and simply-connected complex Lie group* $\widehat{G}$ *corresponding to the complexified Lie algebra* $\widehat{\mathfrak {g}}:=\mathfrak {g}+i\mathfrak {g}$ (*and so* $G/\varGamma$ *is an orbit in* $\widehat{G}/\varGamma$). *Moreover*, *the Levi-factor of* $\widehat{G}$ *is equal to the Levi-factor of* *G*, *i.e.*, $\widehat{S}=S$.

#### Proof

Let $\widehat{G}=\widehat{R}\rtimes \widehat{S}$ and $G=R\rtimes S$ be Levi-decompositions of $\widehat{G}$ and *G*, respectively. Since *R* is connected and simply-connected then, as remarked above, it is closed in its complexification $\widehat{R}$. On the other hand, since $\mathfrak {m}\lhd \mathfrak {g}$ then by the linearity of Lie brackets we deduce that $\mathfrak {m}\lhd \widehat{\mathfrak {g}}$. Thus, $\mathfrak {m}\cap \widehat{\mathfrak {s}}$ is a complex semisimple ideal of $\widehat{\mathfrak {s}}$. However, the complex semisimple Lie algebra $\widehat{\mathfrak {s}}/\mathfrak {m}\cap \widehat{\mathfrak {s}}$ is a subalgebra of the complex Lie algebra $\widehat{\mathfrak {g}}/\mathfrak {m}$. The latter has dimension less than or equal two. Hence, $\dim _{\mathbb {C}}\widehat{\mathfrak {s}}/\mathfrak {m}\cap \widehat{\mathfrak {s}}\le 2$, i.e., $\widehat{\mathfrak {s}}=\mathfrak {m}\cap \widehat{\mathfrak {s}}$ since the smallest complex (non-Abelian) simple Lie algebra is $\mathfrak {sl}_{2}(\mathbb {C})$ and it has dimension 3. We have $\mathfrak {s}=\mathfrak {m}\cap \widehat{\mathfrak {s}}= \widehat{\mathfrak {s}}$, and consequently, *G* is a closed subgroup of $\widehat{G}$ and we can consider the orbits, $\varSigma =G/\varGamma \hookrightarrow \widehat{G}/\varGamma =:X$. $\square$

Clearly, if $\varSigma =G/\varGamma$ has codimension 1 or 2, then *G* cannot be semisimple. In higher codimensions, it is no longer true since, for instance, we have the compact real forms of complex semisimple Lie groups.

#### Reduction to the solvable case

As we will see in Sect. 5 that if $\widehat{G}$ acts on a projective manifold then the radical orbits are closed, see Lemma 5.2. Thus, in the following theorem, we restrict ourselves to complex groups with no non-trivial projective representation, and we prove that the radical orbits are closed (see [20, Theorem 2] and [21, Proposition 2.10] for a general result). Therefore, the first building block will be the compact base of the radical fibration.

##### Proposition 4.1

(Radical-Fibration) *Let* $X=\widehat{G}/\varGamma$ *be a homogeneous parallelizable complex manifold*, *where* $\widehat{G}$ *is a connected and simply-connected complex Lie group*, *and* $\varGamma$ *is a discrete subgroup. Let* $\widehat{G}=\widehat{S}\ltimes \widehat{R}$ *be a Levi-decomposition. Assume there is no non-trivial projective representations of* $\widehat{G}$. *Then the* $\widehat{R}$-*orbits are closed*, *and we have the following fibration.*

2 $$\begin{aligned} \pi \;:\;\widehat{G}/\varGamma \;\xrightarrow { \widehat{R}/\widehat{R}\cap \varGamma } \;\widehat{G}/\widehat{R}\varGamma \;=\; \widehat{S}/\varLambda , \end{aligned}$$

*where* $\varLambda := \widehat{S}\cap \widehat{R}\varGamma$. *Hence*, *in codimension one or two*, *the base* $\widehat{S}/\varLambda = \pi (\varSigma )$ *of the radical fibration* (2) *is a compact complex manifold.*

##### Proof

If $\widehat{G}$ is solvable then the proposition follows. Suppose now that $\widehat{G}$ is not solvable. If $\widehat{R}\varGamma$ is a closed subgroup of $\widehat{G}$, we are done. Assume otherwise, that is the Lie subgroup $\widehat{R}\varGamma$ of $\widehat{G}$ is not closed.

**Claim:** There exists a closed connected solvable complex Lie subgroup $\widehat{H}$ of $\widehat{G}$ containing the connected component $I^{0}_{1}$ of the closure $I_{1}:=\mathrm {cl}_{\widehat{G}}(\widehat{R}\varGamma )$.

**Proof of Claim:** It follows from Zassenhaus Lemma [5, Proposition 2] that $I_{1}^{0}$ is solvable, but of course not necessarily complex. Nevertheless, let $I_{2}$ be the connected complex subgroup of $\widehat{G}$ corresponding to the complexified Lie algebra of $I_{1}$ and consider the connected component $I_{2}^{0}$ and note that it is solvable. Repeat the process and note that since $\widehat{G}$ is not solvable and it has a finite dimension, there exists a proper connected closed solvable complex Lie subgroup $I_{n}=:\widehat{H}$ that contains $I_{1}^{0}\supset \widehat{R}$, as desired.

Now, by our assumption, the homogeneous space $\widehat{G}/N_{\widehat{G}}(\widehat{H})$ is trivial as it is a projective manifold. That means, $N_{\widehat{G}}(\widehat{H})=\widehat{G}$, i.e., $\widehat{H}$ is a connected normal complex solvable subgroup of $\widehat{G}$, hence $\widehat{H}=\widehat{R}\not =\widehat{G}$. This implies $I^{0}_{1}=\widehat{R}$ and hence $I_{1}=\widehat{R}\varGamma$, which contradicts our assumption, i.e., after all, $\widehat{R}\varGamma$ is a closed subgroup of $\widehat{G}$. $\square$

By Theorem 4.1, $S=\widehat{S}$ and, as a consequence, the *S*-orbit through the base point is complex and it lies in $\varSigma =G/\varGamma$. Thus, the *S*-orbit lies in the leaf through the base point. Hence, in the induced radical fibration of $\varSigma$ we have,

$$\begin{aligned} G/\varGamma \xrightarrow {\quad R/R\cap \varGamma \quad } G/R\varGamma \; =\; \widehat{S}/\varLambda \end{aligned}$$

and $\varSigma$ maps onto the base $\widehat{S}/\varLambda$ implying this base is compact. By the Codimension Lemma 2.1, $\mathrm {codim}\; R/R\cap \varGamma =\mathrm {codim}\;\varSigma$. Hence we only have to study Levi-foliation when *G* is solvable with more emphasis on dense Levi-foliation.

#### Reduction to the nilpotent case

The following theorem can be found in [29, Theorem in §5] and [30, Theorem 4.1].

##### Lemma 4.1

(Mostow fibration) *With the above notation*, *let* *G* *and* $\widehat{G}$ *be solvable Lie groups*, *and let* *N* *and* $\widehat{N}$ *be their nilradicals. Then*, *the* *N*-*orbits* (*resp. the* $\widehat{N}$-*orbits*) *in* $\varSigma$ (*resp. in* *X*) *are closed and therefore we can consider the following commutative diagram of nilmanifold-bundles**with the right vertical arrow being holomorphic.*

Note that, the base $G/N\varGamma$ of the Mostow fibration is an Abelian Lie group as *N* contains the commutator subgroup of *G*. But since we have already discussed in Sect. 4.1 the Levi-foliation on Abelian Lie groups, we shall then focus our attention on Levi-foliation on the fiber of the Mostow fibration, i.e., we shall next study the Levi-foliation on compact parallelizable nilmanifolds.

Suppose $\widehat{G}$ is a connected, simply-connected nilpotent complex Lie group. Since the exponential map $\exp :\widehat{\mathfrak {g}}\rightarrow \widehat{G}$ is one-to-one and onto, for any Lie subgroup $\widehat{H}$ of $\widehat{G}$ we can define its *complex hull* $\langle \widehat{H}\rangle _{\widehat{G}}$ to be the smallest connected complex subgroup of $\widehat{G}$ containing $\widehat{H}$. Now assume $\varGamma$ is a discrete subgroup of $\widehat{G}$ with $\langle \varGamma \rangle _{\widehat{G}}=\widehat{G}$. Then it was shown in [18, Theorem 4], using ideas of Barth–Otte [7] that the center $\widehat{Z}$ of $\widehat{G}$ has closed orbits in $\widehat{G}/\varGamma$.

##### Definition 4.1

(*Abelian Group Tower*) An Abelian Lie group (resp. Cousin group) tower of length one is an Abelian complex Lie group (resp. Cousin group). An Abelian group (resp. Cousin group) tower of length $n>1$ is an Abelian complex Lie group (resp. Cousin group) bundle over an Abelian complex Lie group (resp. Cousin group) tower of length $n-1$.

**Fig. 1:**
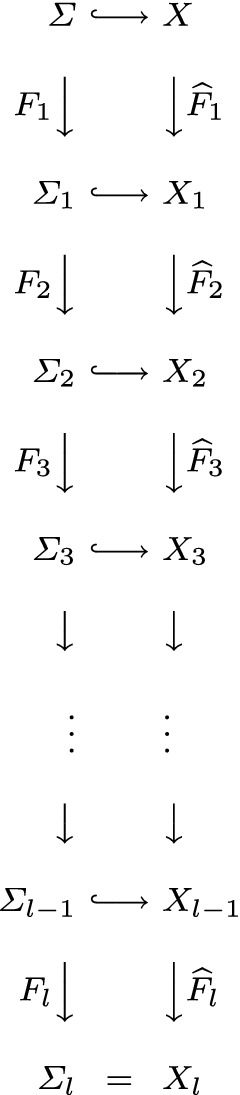
Cousin Tower (CT)

Let us further suppose that $\varSigma =G/\varGamma$ is a generic homogeneous CR-manifold of codimension *k* with globalization $X=\widehat{G}/\varGamma$, where $\widehat{G}$ is connected, simply-connected, nilpotent complex Lie group. Assume as well that the leaves of the Levi-foliation are *dense*. Then $\mathcal {O}(X)=\mathbb {C}$, the reason is that the restriction of any holomorphic function $f\in \mathcal {O}(X)$ to the compact manifold $\varSigma$ attains its maximum, say at the point $y\in \varSigma$. Now, let $\mathcal {L}$ be the leaf through *y* and note that, by the maximum principle, $f|_{\mathcal {L}}$ is constant which in turn implies that $f|_{\varSigma }$ is also constant since $\mathcal {L}$ is dense in $\varSigma$. Recall that, locally $\varSigma = \varDelta _{n-k}\times \mathrm {Re}\; \varDelta _{k}$ and $X= \varDelta _{n-k}\times \varDelta _{k}$ where $\varDelta _{j}$ is a polydisk in $\mathbb {C}^{j}$. Thus, *f* itself must be constant, for a holomorphic function cannot be non-constant in only one real part. As a consequence, it follows that, $\langle \varGamma \rangle _{\widehat{G}}=\widehat{G}$. In the latter setting it is known, see [31] or [4], that $\widehat{G}/\varGamma$ is a Cousin tower. There is an induced fibration of $\varSigma$, see Fig. 1. Two cases can occur at each step of the tower: if $\widehat{F}_{j}$ is a non-compact Cousin group of codimension $n_{j}$, then there exists a holomorphic fibration, see e.g., [1] or [40],

$$\begin{aligned} \widehat{F}_{j}\;\xrightarrow { (\mathbb {C}^{*})^{n_{j}}} \;T_{j} \end{aligned}$$

and an induced fibration of the real fiber

$$\begin{aligned} F_{j}\;\xrightarrow { (S^{1})^{n_{j}}}\; T_{j}. \end{aligned}$$

If $\widehat{F}_{j}$ is compact, then $F_{j}=\widehat{F}_{j}$ is a complex torus (here set $n_{j}=0$). For dimension reasons, we then have

$$\begin{aligned} \sum \limits _{j=1}^{l} \; n_{j}\; =\; k. \end{aligned}$$

Furthermore, the leaves of the Levi-foliation on $\varSigma$ induce a Levi-foliation on each fiber $F_{j}$ in the Cousin tower where each leaf $\mathcal {L}_{F_{j}}$ of this induced foliation can be expressed as the covering

$$\begin{aligned} \mathcal {L}_{F_{j}}\; \xrightarrow {(\mathbb {Z})^{n_{j}}}\; T_{j}. \end{aligned}$$

The following figure summarizes the obtained reductions in this chapter [$\varSigma _{l}$ denotes the base of the radical fibration (2)].

### Main theorem for parallelizable manifolds

We first remark that the leaves of the Levi-foliation do not have to be dense or compact in $\varSigma$. Nonetheless, the discussion in the previous subsections shows that, in all cases, one can reduce to the case where $\widehat{G}$ is nilpotent. Furthermore, since $\varSigma$ is generic in *X*, then $\langle \varGamma \rangle _{\widehat{G}}=\widehat{G}$ and by a result in [7], the center orbits are closed. Hence, one can always reduce to the Abelian tower as in Fig. 1, where the complex fibers $\widehat{F}_{j}$ may have a product of $\mathbb {C}^{*}$. The following table sets out the situations that would arise in codimension one and two.

**Table 1:** (CT notations)

Cousin Tower ($\mathrm {CT}$) of parallelizable complex homogeneous manifolds *X*			
Name	Description	Notation	Corresp. circle tower for $\varSigma$
Torus tower	the tower has **no** Cousin groups of positive codimension	$\mathrm {CT}_{0}$	$\mathrm {CT}_{0}$ or $\mathrm {ct}_{0}$
Cousin tower of codim. 1	the tower has **one** Cousin group of codim. 1	$\mathrm {CT}_{1}$	$\mathrm {ct}_{1}$
Cousin tower of codim. 2 and 1 step	the tower has **one** Cousin group of codim. 2	$\mathrm {CT}_{2}$	$\mathrm {ct}_{2}$
Cousin tower of codim. 2 and 2 steps	the tower has **two** Cousin groups of codim. 1. See Example 4.4	$\mathrm {CT}_{1,1}$	$\mathrm {ct}_{1,1}$
Torus tower with one $\mathbb {C}^{*}$	the tower has one $\mathbb {C}^{*}$ but **no** Cousin groups of positive codimension	$\mathrm {CT}^{*}_{0}$	$\mathrm {ct}^{*}_{0}$
Torus tower with two $$\mathbb {C}^{*}$$’s	the tower has two $$\mathbb {C}^{*}$$’s but **no** Cousin groups of positive codimension	$$\mathrm {CT}^{**}_{0}$$	$$\mathrm {ct}^{**}_{0}$$
Abelian tower of one Cousin group of codim. 1 and one $\mathbb {C}^{*}$	the tower has **one** Cousin group of codim.1 and one $\mathbb {C}^{*}$	$\mathrm {CT}^{*}_{1}$	$\mathrm {ct}^{*}_{1}$

We summarize the preceding discussion in the following theorem.

#### Theorem 4.2

(Main Theorem in Sect. 4) *Let* *G* *be a connected and simply-connected Lie group and* $\varGamma$ *be a discrete subgroup of* *G*. *Let further* $G = S\ltimes R$ *be a Levi-decomposition of* *G* *and* $\mathfrak {r}$ *be the Lie algebra of the radical* *R*. *Suppose that* $\varSigma :=G/\varGamma$ *is a compact*, *Leviflat*, *parallelizable*, *homogeneous CR-manifold of codimension one or two. Then*, *any semisimple-factor* *S* *is a complex semisimple Lie group and* *G* *is a closed subgroup of the connected and simply-connected complex Lie group* $\widehat{G}:=S\ltimes \widehat{R},$ *where* $\widehat{R}$ *is the connected and simply-connected complex solvable Lie group corresponding of the complexified Lie algebra* $\widehat{\mathfrak {r}}:=\mathfrak {r}+i\mathfrak {r}$. *Hence*, $\varSigma$ *is a compact generic* *G*-*orbit in the parallelizable complex orbit* $X:= \widehat{G}/\varGamma$. *Furthermore*, $\widehat{R}$-*orbits are closed in* *X* *and hence* *R*-*orbits are compact*, *and in the CT tower* (1), *one has*:

- *If the Levi-foliation is dense* [*i.e.*, *iff* $\mathcal {O}(X)=\mathbb {C}],$ *then in codimension*
  1. *one*: $$\varSigma =\mathrm {ct}_{1}\subset X=\mathrm {CT}_{1},$$
  2. *two*: *either* $\varSigma =\mathrm {ct}_{2}\subset X=\mathrm {CT}_{2},$ *or* $\varSigma =\mathrm {ct}_{1,1}\subset X=\mathrm {CT}_{1,1}$.
- *If the Levi-foliation is compact*, *then in codimension*
  1. *one*: $$\varSigma =\mathrm {ct}^{*}_{0}\subset X=\mathrm {CT}^{*}_{0},$$
  2. *two*: $$\varSigma =\mathrm {ct}^{**}_{0}\subset X=\mathrm {CT}^{**}_{0}.$$
- *If the foliation is neither compact nor dense*, *then* $\varSigma =\mathrm {ct}^{*}_{1}\subset X=\mathrm {CT}^{*}_{1}$.

### A non-trivial Cousin group bundle over a Cousin group

Let $$M:=\begin{pmatrix}1&{} -2\\ 1&{}1\end{pmatrix}\in \mathrm {GL}_{n}(\mathbb {C})$$ and let $B:=\mathbb {C}^{2}$. Then, *M* acts on *B* as a linear transformation. On the other hand, *M* is similar to a diagonal matrix $\gamma$ with diagonal entries its eigenvalues—in no particular order $\lambda _{1}:=1+i\sqrt{2}$ and $\lambda _{2}:=1-i\sqrt{2}$. Say, *M* is similar to $$\gamma :=\begin{pmatrix}\lambda _{1}&{}0\\ 0&{}\lambda _{2} \end{pmatrix}$$. Rewrite $\lambda _{1}$ and $\lambda _{2}$ as $\sqrt{3} {\text {e}}^{i\theta }$ and $\sqrt{3} {\text {e}}^{-i\theta }$ , respectively, where $\cos \theta =\frac{1}{\sqrt{3}}$ and $\sin \theta = \frac{\sqrt{2}}{\sqrt{3}}$. Let $A:=\mathbb {C}^{*}\times \mathbb {C}^{*}$ and write it as the diagonal group $$A=\left\{ \begin{pmatrix}\alpha &{}0 \\ 0&{}\beta \end{pmatrix}; \ \alpha ,\beta \in \mathbb {C}^{*}\right\} $$. Define the lattice $$\varGamma _{A}\;:=\;\left\langle \gamma ^{n}\right\rangle _{n\in \mathbb {Z}} \;= \;\left\{ \sqrt{3^{n}}\;\begin{pmatrix} {\text {e}}^{in\theta }&{}0\\ 0&{}{\text {e}}^{-in\theta }\end{pmatrix}; \; \; n\;\in \;\mathbb {Z}\right\} $$ of *A*. Note that its pullback to the universal covering of *A* has rank 3.

**Claim:** The complex Abelian group $A/\varGamma _{A}$ is a Cousin group.

**Proof of Claim:** Note that the kernel of the holomorphic homomorphism $\mathbb {C}^{2}\rightarrow \mathbb {C}^{*}\times \mathbb {C}^{*}; (z,w)\mapsto ({\text {e}}^{2\pi iz}, {\text {e}}^{2\pi iw})$ is $\left\{ (1 ,0), (0,1)\right\} _{\mathbb {Z}}$. Hence,

$$\begin{aligned} A/\varGamma _{A}\; \cong \; \mathbb {C}^{2}\big {/}\big \{(1,0), (0,1), (\gamma _{1},\gamma _{2})\big \}_{\mathbb {Z}}, \end{aligned}$$

where (choose the branch cut to be along the positive real axis $\mathbb {R}^{\ge 0}$)

$$\begin{aligned} \gamma _{1}:= & {} \log (\lambda _{1})= \ln \sqrt{3}+i\theta , \\ \gamma _{2}:= & {} \log (\lambda _{2})= \ln \sqrt{3}+i(2\pi -\theta ). \end{aligned}$$

Thus, it is enough to show the lattice $\left\{ (1 ,0), (0,1),(\gamma _{1},\gamma _{2})\right\} _{\mathbb {Z}}$ satisfies the irrationality Condition (see, e.g., [1]), i.e., the slope $\frac{\theta }{2\pi -\theta }\not \in \mathbb {Q}$. Let us therefore suppose, to the contrary, $\frac{\theta }{2\pi -\theta }=q$ for some $q\in \mathbb {Q}\setminus \{0\}$. Then $\theta =\frac{m}{n}\pi$ for some relatively prime integers $m,n\in \mathbb {Z}\setminus \{0\}$. Hence,

$$\begin{aligned} \dfrac{1}{\sqrt{3}}+i\dfrac{\sqrt{2}}{\sqrt{3}}=\cos \theta +i\sin \theta ={\text {e}}^{i\theta } \end{aligned}$$

and since $n\theta =m\pi$, then one has

$$\begin{aligned} \left( \dfrac{1}{\sqrt{3}}+i\dfrac{\sqrt{2}}{\sqrt{3}}\right) ^{n} ={\text {e}}^{in\theta }\in \mathbb {R},\quad \text {i.e.,}\;\;\left( 1+i\sqrt{2}\right) ^{n}\in \mathbb {R}. \end{aligned}$$

Thus,

$$\begin{aligned} \sqrt{2} i\left\{ \left( {\begin{array}{c}n\\ 1\end{array}}\right) -2 \left( {\begin{array}{c}n\\ 3\end{array}}\right) +4 \left( {\begin{array}{c}n\\ 5\end{array}}\right) -\cdots + (-1)^{k}2^{k} \left( {\begin{array}{c}n\\ 2k+1\end{array}}\right) +\cdots \right\} =0 \; \; \ldots \; \; (*) \end{aligned}$$

We note that *n* cannot be odd since the summation would be an odd integer. On the other hand, if *n* is even, say $n=2^{p}q$ for an odd integer *q*, then $2^{p}$ would divide $$\left( {\begin{array}{c}2^{p}q\\ 2k+1\end{array}}\right) $$. Indeed, since

$$\begin{aligned} \left( {\begin{array}{c}n\\ m\end{array}}\right) = \dfrac{n(n-1)!}{m(m-1)!(n-m)!}=\dfrac{n}{m}\left( {\begin{array}{c}n-1\\ m-1\end{array}}\right) \end{aligned}$$

then, if $n=2^{p}q$ and $m=2k+1$, one has

$$\begin{aligned} (2k+1)\left( {\begin{array}{c}n\\ 2k+1\end{array}}\right) =2^{p}q\left( {\begin{array}{c}n-1\\ 2k\end{array}}\right) . \end{aligned}$$

Therefore,

$$\begin{aligned} 2^{p}\mid \left( {\begin{array}{c}n\\ 2k+1\end{array}}\right) . \end{aligned}$$

Hence, the equation $(*)$ can be written as $q+2t =0$ for some integer *t*. But *q* is an odd integer. This is a contradiction, and the Claim is proved.

Now consider the Cousin group $B/\varGamma _{B}$, where $\varGamma _{B}:=\left\{ (1,0),(0,1),(i,i\sqrt{2})\right\}$. Let also $P\in \mathrm {GL}_{2}(\mathbb {C})$ be a matrix such that $M=P^{-1}\gamma P$ and define the solvable (non-Abelian) complex Lie group $G:=A\ltimes _{M} B$ by

$$\begin{aligned} (A_{1},b_{1})\cdot (A_{2},b_{2})\;:=\; \Big (A_{1}A_{2},\; (P^{-1}A_{1}P) b_{2}+b_{1}\Big ). \end{aligned}$$

Define the discrete subgroup

$$\begin{aligned} \varGamma :=\varGamma _{A}\ltimes _{M} \varGamma _{B}=\Big \{ \big (\psi ,\; M^{n} \omega _{1}+\omega _{2}\big )\;\Big {|}\;\; \psi \in \varGamma _{A},\; \omega _{1},\omega _{2}\in \varGamma _{B},\; n\in \mathbb {Z}\Big \}. \end{aligned}$$

Consider the closed subgroup

$$\begin{aligned} H:=\varGamma _{A}\ltimes _{M} B\;=\;\Big \{ \big (\psi ,\; M^{n} b_{1}+b_{2}\big )\; \Big {|}\;\; \psi \in \varGamma _{A},\; b_{1},b_{2}\in B,\; n\in \mathbb {Z}\Big \} \end{aligned}$$

and note that $G/H=A/\varGamma _{A}$ and that $H/\varGamma =B/\varGamma _{B}$. Now, we consider the following principal fiber bundle of Cousin groups

3 $$\begin{aligned} G/\varGamma \xrightarrow { B/\varGamma _{B}} A/\varGamma _{A}. \end{aligned}$$

In particular, the bundle above is not topologically trivial, since otherwise the fundamental group of $G/\varGamma$ would be Abelian, while it is not.

## Projective orbits

In this section, we consider compact homogeneous Leviflat CR-manifolds $\varSigma = G/H$ that are orbits in some complex projective space $\mathbb {P}_{n}$. For convenience throughout this section, we assume that the group *G* admits an *almost faithful* representation into the group of holomorphic automorphisms of the projective space $\mathbb {P}_{n}$ at hand. We warn the reader, however, that in the general setting (treated in the next section) this is not always the case and appropriate modifications must be made.

We first introduce some notation. Let $\widehat{G}$ denote the smallest connected complex Lie group that contains *G*, i.e., the group corresponding to the complexified Lie algebra $\widehat{\mathfrak {g}} := \mathfrak {g} + i \mathfrak {g}$, where $\mathfrak {g}$ is the Lie algebra of *G*. Using Chevalley’s result [14] that the commutator subgroup of $\widehat{G}$ is acting as an algebraic group, we show first that the radical $\widehat{R}$ of $\widehat{G}$ is central. Then we show that the leaves of the Levi-foliation of $\varSigma$ are compact, and thus are biholomorphic to flag manifolds. Finally, when the codimension of $\varSigma$ is one or two, we give the classification.

### Preliminaries

Let $\widehat{G}$ be any complex Lie group and $\widehat{G}=\widehat{S}\ltimes \widehat{R}$ be a Levi-decomposition. Let $\widehat{\mathfrak {g}}$, $\widehat{\mathfrak {s}}$, and $\widehat{\mathfrak {r}}$ be the corresponding complex Lie algebras of the groups $\widehat{G}$, $\widehat{S}$, and $\widehat{R}$, respectively. Recall the following classical fact in the theory of Lie algebras (see e.g., [16]).

#### Lemma 5.1

*Let* $\mathfrak {g}=\mathfrak {s}\ltimes \mathfrak {r}$ *be a Levi-decomposition of a Lie algebra* $\mathfrak {g}$. *Then*, *the ideal* $[\mathfrak {g}, \mathfrak {r}]$ *is nilpotent.*

#### Lemma 5.2

*Consider the nilpotent subalgebras* $\widehat{\mathfrak {n}}:= [\widehat{\mathfrak {g} }, \widehat{\mathfrak {r}}]$ *and* $\mathfrak {n}:=[\mathfrak {g},\mathfrak {r}]$ *of* $\widehat{\mathfrak {g}}$ *and* ${\mathfrak {g}},$ *respectively. Then*,

4 $$\begin{aligned} \widehat{\mathfrak {n}} \; = \; \mathfrak {n} \; + \; i \mathfrak {n}. \end{aligned}$$

*Similarly*, *for any term* $\widehat{\mathfrak {m}}$ *in the descending central series of* $\widehat{\mathfrak {n}},$ *one has*

5 $$\begin{aligned} \widehat{\mathfrak {m}} \; = \; \mathfrak {m} \; + \; i \mathfrak {m}, \end{aligned}$$

*where* $\mathfrak {m}$ *is the corresponding term in the descending central series of* $\mathfrak {n}$.

#### Proof

The inclusion RHS contained in LHS follows since $\mathfrak {n}$ is clearly a nilpotent ideal in $\widehat{\mathfrak {g}}$, so $\mathfrak {n}$ is contained in the nilradical $\widehat{\mathfrak {g}}$. Conversely, consider the Lie algebra homomorphism $\pi :\widehat{\mathfrak {g}}' \rightarrow \widehat{\mathfrak {g}}'/ (\mathfrak {n} + i\mathfrak {n})$. Our proof will be complete if we show that the quotient is semisimple, because this means that $\widehat{\mathfrak {n}}$ is contained in the kernel of $\pi$. In fact, one has,

6 $$\begin{aligned} \widehat{\mathfrak {g}}':= & {} [\mathfrak {g} + i \mathfrak {g}, \mathfrak {g} + i\mathfrak {g}] \nonumber \\= & {} [\mathfrak {g},\mathfrak {g}]+i[\mathfrak {g},\mathfrak {g}]\nonumber \\= & {} [\mathfrak {r},\mathfrak {g}]+\mathfrak {s} + i\left( [\mathfrak {r},\mathfrak {g}] +\mathfrak {s}\right) \nonumber \\= & {} (\mathfrak {s} + i \mathfrak {s}) + (\mathfrak {n} + i \mathfrak {n}) \end{aligned}$$

and the result in (4) follows from this. Similarly, the claim in (5) follows from (4) and the linearity of the Lie brackets. $\square$

The following well-known proposition will often be used in this section. For general notions of linear algebraic groups and their orbits, we refer the reader to [10, 25], or [37].

#### Proposition 5.1

*Let* $X:=G/H$ *be an algebraic homogeneous space of a linear algebraic group* *G* *and let* *N* *be a normal algebraic subgroup of* *G*. *Assume that the action of* *N* *on* *X* *is algebraic*, *then* *N*-*orbits are closed.*

#### Proof

Since *N* acts algebraically, then its orbits are Zariski open in their closures. The boundary of each *N*-orbit consists of *N*-orbits of strictly lower dimension. Since *N* is a normal subgroup of *G*, all orbits have the same dimension and so the complement $\overline{N\cdot x}\setminus N\cdot x$ is empty, i.e., *N*-orbits are closed. $\square$

We recall, furthermore, that an algebraic linear group is called *unipotent* if it is isomorphic to a closed subgroup of the group of upper triangular matrices with 1’s in the diagonal. We also recall the fact that orbits of an algebraic action of a connected unipotent group are algebraic geometrically isomorphic to some affine space $\mathbb {C}^{p}$ (e.g., [9, Theorem 1.4]).

### Main theorem

In the setting of this section, the following is the first main theorem in this chapter.

#### Theorem 5.1

*The radical* $\widehat{R}$ *is central and* $\widehat{G}=\widehat{S}\times \widehat{R}$ (*possibly with finite intersection*). *The real group* *G* *splits accordingly.*

#### Proof

Enough to show that the unipotent subgroup $\widehat{N}:=[\widehat{G},\widehat{R}]$ is trivial, because then by (6) the commutator subgroup $\widehat{G}'=\widehat{S}$ and therefore the result follows.

Recall first that every non-trivial unipotent group has a positive-dimensional center. Thus $\widehat{N}$ is trivial if and only if the last term, say $\widehat{M}$, of the descending central series of $\widehat{N}$ is trivial.

Let us now assume the contrary, that the unipotent Abelian normal subgroup $\widehat{M}$ is not trivial. By the discussion above, the $\widehat{M}$-orbits are closed and biholomorphic to some $\mathbb {C}^{q}$. Thus $\widehat{M}\widehat{H}$ is a closed subgroup of $\widehat{G}$ and therefore we can consider the following fibrations:where $J:=\widehat{M}\widehat{H}\cap G$ and the fiber $F:=J/H$ is a compact submanifold of the complex fiber $\widehat{F}:=\widehat{M}/\widehat{M}\cap \widehat{H}$. In fact, $\widehat{M}$ acts on $\widehat{F}$ with a finite isotropy as it is an Abelian characteristic subgroup of $\widehat{G}$ and the action is almost effective. Say, $\widehat{M}\cap \widehat{H}=:\varLambda$.

Consider now the stabilizer group $I:={\text {Stab}}_{\widehat{M}}(J\cdot x_{0})$ and note that it is a closed subgroup of $\widehat{M}$ since $J\cdot x_{0}$ is a compact submanifold of $\widehat{M}\cdot x_{0}$. Since $\varLambda$ fixes all points of the latter orbit, then $\varLambda \subset I$. This leads to a fibration of the Euclidean space $\mathbb {C}^{k}$ by compact fibers,

$$\begin{aligned} \mathbb {C}^{k}\;=\; \widehat{M}/\varLambda \;\xrightarrow { F=I/\varLambda } \;\widehat{M}/I. \end{aligned}$$

However, by Borel–Serre Theorem [12], such fibration does not exist unless *F* is just a point, i.e., $I=\varLambda$. But since $M\subset I$ then $M=\{e\}$ and hence by Lemma 5.2, we have $\widehat{M}=\{e\}$. From this contradiction, we conclude that $\widehat{N}$ has to be trivial. $\square$

### Three basic fibrations

The goal is to prove that in the projective case $\mathcal {L}$ is homogeneous rational, i.e., a flag manifold. In fact, we need only to show that $\mathcal {L}$ is compact because a compact complex homogeneous Kähler manifold is a product of a compact complex torus and a flag manifold (see [11]). However, by the Borel Fixed Point Theorem, no positive-dimensional compact complex torus can be embedded equivariantly in $\mathbb {P}_{n}$ (see e.g., [10]). We follow the notation of the paper where we know that $\widehat{G}=\widehat{S}\times \widehat{R}$ with possible finite intersection and where $\widehat{R}$ is central in $\widehat{G}$. The real group *G* splits accordingly.

#### Theorem 5.2

*The leaf* $\mathcal {L}$ *is a flag manifold.*

#### Proof

If $\mathcal {L}$ is trivial, then there is nothing to prove, so assume $\mathcal {L}$ is not trivial. There are three cases in the proof which goes (except for the last subcase) by induction on the codimension *k* of $\mathcal {L}$ in $\varSigma$. The case of $k=0$ is clear.

**The case where** ${{\widehat{S}}}$ **does not act transitively on** ${{X}}$.

Since $\widehat{S}$ is semisimple and is therefore acting algebraically, we have the quotient $\pi : X\rightarrow X/\widehat{S}$ the base of which is a Stein Abelian group (this is the commutator fibration $\widehat{G}/\widehat{H}\rightarrow \widehat{G}/\widehat{G}'\widehat{H}$ and the base is a Stein Abelian group, see [23]). The image of $\varSigma$ is an *R*-orbit which is a totally real product of circles. In particular, $\mathcal {L}$ is contained and of lower codimension in the neutral fiber $\pi ^{-1}(\pi (x_{0}))$. Applying the induction assumption to this fiber yields the desired result.

**The case where** ${{\widehat{S}}}$ **acts transitively on** ${{X}}$ **and** ${{\widehat{R}\not =\{e\}}}$.

Since $\widehat{S}$ is acting algebraically, *X* is Zariski open in its closure *Y*. Since $\widehat{R}$ is acting linearly and stabilizes *X*, it follows that it stabilizes *Y*. Thus if $E := Y \setminus X$ is the boundary, it follows that $\widehat{R}$ is in the stabilizer $\widehat{L}$ of *E* in the stabilizer of *Y* in the linear group. The main point is that $\widehat{L}$ is an algebraic group acting on *X*. It then follows that $\widehat{R}$ is contained in the centralizer $\widehat{Z}$ of $\widehat{S}$ in $\widehat{L}$ which is also an algebraic group acting on *X*. Thus, we consider the quotient $\pi : X\rightarrow X/\widehat{Z}$. Since the $\pi$-fibers are isomorphic to manifolds of the form $\mathbb {C}^{s}\times (\mathbb {C}^{*})^{t}$, the intersections of these fibers with $\mathcal {L}$, which are complex analytic sets contained in compact subsets, are discrete. Thus $\pi (\mathcal {L})=:Q$ is of lower codimension in $\pi (\varSigma )$ and induction implies that *Q* is homogeneous rational. Since $\mathcal {L} \rightarrow Q$ is $\widehat{G_{\mathcal {L}}}$-equivariant, it is a covering map which is injective, because *Q* is simply-connected. Then the result follows by induction hypothesis. We also conclude from the preceding discussion that the *algebraic hull* of $\widehat{R}$ is an Abelian algebraic group which stabilizes *X*.

**The case where** ${{\widehat{G}=\widehat{S}}}$.

Since $\widehat{S}$ acts algebraically, the fundamental group of *X* is finite. Thus, replacing $\widehat{H}$ by $H\widehat{H}^{\circ}$ (i.e., the minimality condition introduced in Sect. 3.2) only entails going to a finite cover where $\widehat{S}$ still acts algebraically. Define $\widehat{N}$ to be the open subgroup of the normalizer of $\widehat{G_{\mathcal {L}}}^{\circ}$ so that $\widehat{N}/\widehat{H}$ is connected.

$\bullet$ If $\widehat{G_{\mathcal {L}}}^{\circ}$ is not normal in $\widehat{S}$. Then, $\mathcal {L}$ is contained and of lower codimension in the neutral fiber of the fibration $X\rightarrow X/\widehat{N}$. Applying the induction assumption to this fiber yields the desired result.

$\bullet$ If $\widehat{G_{\mathcal {L}}}^{\circ}$ is normal in $\widehat{S}$. Then, $\widehat{G_{\mathcal {L}}}^{\circ}$ is semisimple which is acting algebraically. Since its orbits are therefore Zariski open in their closures, it follows that $\mathcal {L}$ is closed in this case, and the result straightforwardly follows. $\square$

#### Leaf-reduction

Taking $\widehat{H}=H\widehat{H}^{\circ}$ as above and observing that the stabilizer in $\widehat{G}$ of the compact variety $\mathcal {L}$ is the closed complex subgroup $\widehat{G_{\mathcal {L}}}=\widehat{H}\widehat{S}_{1}$ , we have the leaf-reduction

7 $$\begin{aligned} \widehat{G}/\widehat{H}\xrightarrow {\mathcal {L}} \widehat{G}/\widehat{G_{\mathcal {L}}}. \end{aligned}$$

If our focus is on $\varSigma$, the restriction of this map to $\varSigma$ is its *G*-equivariant CR-holomorphic leaf on to a totally real (generic) hypersurface *G*/*J* in $\widehat{G}/\widehat{G_{\mathcal {L}}}$, where $J:=\widehat{G_{\mathcal {L}}}\cap G$. Note that, $\dim _{\mathbb {R}} G/J=\mathrm {codim} \; \varSigma$ (see Lemma 2.1).

### Classification for codimension one and two

#### The radical orbits

The aim of this subsection is to prove the following proposition.

##### Proposition 5.2

*Suppose* $\widehat{S}$ *is not transitive on* *X*; 

1. *If* $$\mathrm {codim}\; \varSigma =1,$$ *then* $$\varSigma = Q \times S^{1} \subset X = Q \times \mathbb {C}^{*} $$.
2. *If* $\mathrm {codim}\; \varSigma =2,$ *then* $\varSigma = Q \times (S^{1})^{2}\subset X$ *where* $X=Q \times (\mathbb {C}^{*})^{2}$ *or* $X= Q \times \mathbb {P}_{1}\times \mathbb {C}^{*}$.

The proof is based on the following lemma which is true for any codimension.

##### Lemma 5.3

*Let* $\widehat{N}$ *be the normalizer of the connected component* $\widehat{H}^{\circ}$ *in* $\widehat{G}$. *Suppose that* $\widehat{S}$-*orbits are compact. Then*, $\widehat{N}=\widehat{R}\widehat{H}$ *and the radical fibration is holomorphically trivial.*

##### Proof

Consider the normalizer fibration $\widehat{G}/\widehat{H}\xrightarrow {\; \widehat{N}/\widehat{H}\; } \widehat{G}/\widehat{N}$. Restrict this bundle to the $\widehat{S}$-orbit to obtain the fibration $\widehat{S}/(\widehat{S}\cap \widehat{H})\longrightarrow \widehat{S}/(\widehat{S}\cap \widehat{N})$. Since $\widehat{S}/(\widehat{S}\cap \widehat{H})$ is a flag manifold then $\widehat{S}\cap \widehat{H}$ is connected, and thus it is contained in $\widehat{H}^{0}$. Therefore $\widehat{S}\cap \widehat{N}$ normalizes the parabolic group $\widehat{S}\cap \widehat{H}$ and hence $\widehat{S}\cap \widehat{H}=\widehat{S}\cap \widehat{N}$. Now since $\widehat{R}$ is central then $\widehat{R}\subset \widehat{N}$ and the above shows that $\widehat{R}/(\widehat{R}\cap \widehat{H})=\widehat{N}/\widehat{H}$. Furthermore, the $\widehat{S}$-orbits form holomorphic sections of the radical fibration $X=\widehat{G}/\widehat{H}\rightarrow \widehat{G}/\widehat{R} \widehat{H}$. Thus, this fibration is holomorphically trivial, i.e., $X\cong \widehat{R}/(\widehat{R}\cap \widehat{H})\times \widehat{S}/(\widehat{S}\cap \widehat{H})$. $\square$

Now we turn to the proof of Proposition 5.2.

##### Proof

Since the base of the commutator fibration $\widehat{G}/\widehat{H} \xrightarrow {\widehat{S}/(\widehat{S}\cap \widehat{H})} \widehat{G}/\widehat{S}\widehat{H}$ is a Stein Abelian group, then we have (see Lemma 2.1) the following:

**In codimension one**, $\widehat{G}/\widehat{S}\widehat{H}=\mathbb {C}^{*}$ and $\widehat{S}/(\widehat{S}\cap \widehat{H})=\mathcal {L}$. Thus, the $\widehat{S}$-orbits are compact and one applies Lemma 5.3.

**In codimension two**, either $\widehat{G}/\widehat{S}\widehat{H}=\mathbb {C}^{*}\times \mathbb {C}^{*}$ and $\widehat{S}/(\widehat{S}\cap \widehat{H})=\mathcal {L}$, or $\widehat{G}/\widehat{S}\widehat{H}=\mathbb {C}^{*}$ and $\widehat{S}/(\widehat{S}\cap \widehat{H})=\mathcal {L}\times \mathbb {P}_{1}$ (see e.g., Lemma 5.6). Thus, the $\widehat{S}$-orbits are compact in either case and one can apply Lemma 5.3 again. $\square$

#### Classification of the leaf-spaces

To give a classification in codimension one and two, we need to establish some essential facts. By Proposition 5.2, the only remaining case to consider is when $\widehat{G}=\widehat{S}$. Also, we impose the minimality condition, i.e., replace $\widehat{H}$ by $H\widehat{H}^{\circ}$ as discussed in Sect. 3.2. Recall that, this assumption only entails going to a finite cover where $\widehat{S}$ still acts algebraically. Moreover, consider the leaf-reductions defined in Sect. 5.3.1.

8 $$\begin{aligned} \widehat{S}/\widehat{H}\xrightarrow {\mathcal {L}} \widehat{S}/\widehat{G_{\mathcal {L}}}=:Z \end{aligned}$$

and the induced reduction for $\varSigma$

$$\begin{aligned} S/ H\xrightarrow {\mathcal {L}} S/J=:Y. \end{aligned}$$

We recall that the *real leaf-space* Y is a totally real homogeneous CR-submanifold of the *complex leaf-space* *Z* and therefore $\dim _{\mathbb {R}} Y=\dim _{\mathbb {C}}Z=\mathrm {codim}\; \varSigma$. Recall also that the maximal connected complex subgroup *M* of *S* is contained in *J* (see Corollary 3.3). Thus, we have the following lemma.

##### Lemma 5.4

*The real leaf-space* *Y* *is an orbit of a real form of* $\widehat{S}$.

##### Lemma 5.5

*If* *Z* *is compact*, *then the real form* *S* *of* $\widehat{S}$ *cannot be compact.*

##### Proof

If *Z* is compact then it is simply-connected. By Montgomery Theorem [27], the compact form of $\widehat{S}$ acts transitively on *Z*. But this is not possible since the codimension is assumed to be positive. $\square$

Since the only one-dimensional complex homogeneous manifold of a complex semisimple Lie group is $\mathbb {P}_{1}$, it follows that we have the following classification theorem when $\varSigma =S/H$ has codimension one in $X=\widehat{S}/\widehat{H}$.

##### Theorem 5.3

*In codimension one*, $\varSigma = Q\times S^{1}$ *and* $X=Q\times \mathbb {P}_{1},$ *where* $Q=\mathcal {L}$ *is a flag manifold.*

##### Proof

The complex leaf-space *Z* is one-dimensional, hence $Z=\mathbb {P}_{1}$ as a holomorphic orbit of $\mathrm {SL}_{2}(\mathbb {C})$ and $Y=S^{1}$ as a corresponding orbit of $\mathrm {SL}_{2}(\mathbb {R})$. The proof that the leaf-reduction is trivial follows by Lemma 5.6. $\square$

Note that the minimality condition here is not necessary since *X* is simply-connected.

Now we turn our attention to *two-dimensional* leaf-spaces. Assume first that the complex leaf-space *Z* is compact and recall that the only two-dimensional flag manifolds are $\mathbb {P}_{1}\times \mathbb {P}_{1}$ and $\mathbb {P}_{2}$ with complex Lie groups $\mathrm {SL}_{2}(\mathbb {C})\times \mathrm {SL}_{2}(\mathbb {C})$ and $\mathrm {SL}_{3}(\mathbb {C})$, respectively. (For the complete list of compact complex homogeneous surfaces see [39].) Recall also (see [28]) the only compact real homogeneous surfaces are

1. orientable surfaces: $S^{1}\times S^{1}$, and $S^{2},$
2. non-orientable surfaces: $\mathbb {RP}_{2}$, and the Klein bottle.

Combining these facts, we have the following proposition.

##### Proposition 5.3

(Two-dimensional compact leaf-spaces) *In codimension two. If the leaf-space* *Z* *is compact*, *then either*

1. $Z=\mathbb {P}_{1}\times \mathbb {P}_{1}$. *In this case*, $\widehat{S}=\mathrm {SL}_{2}(\mathbb {C})\times \mathrm {SL}_{2}(\mathbb {C})$ *and the real leaf-space* *Y* *is orientable and isomorphic to*
  (i) $S^{1}\times S^{1}$ *as an orbit of the non-compact real form* $\mathrm {SL}_{2}(\mathbb {R})\times \mathrm {SL}_{2}(\mathbb {R}),$
  (ii) *the* 2-*dimensional closed orbit* $\mathcal {M}_{1}$ *of the real form* $\mathrm {SL}_{2}(\mathbb {C})$ (*embedded as an antiholomorphic diagonal in* $\widehat{S}$) *acting by the antiholomorphically twisted diagonal embedding* $A\mapsto (A,(\bar{A}^{-1})^{t})$ *in* $\widehat{S}$. *As manifolds*, $\mathcal {M}_{1}\cong S^{2}$.
2. $Z=\mathbb {P}_{2}$. *In this case*, *the real leaf-space* *Y* *is non-orientable and isomorphic to* $\mathbb {RP}_{2}$ *as an orbit of the non-compact real form* $\mathrm {SL}_{3}(\mathbb {R})$ *of the simple complex Lie group* $\mathrm {SL}_{3}(\mathbb {C})$.

##### Proof

By Lemma 5.5, we only have to check the non-compact real forms of the semisimple complex Lie groups.

1. If $Z=\mathbb {P}_{1}\times \mathbb {P}_{1}$, then the complex Lie group of this surface is $\mathrm {SL}_{2}(\mathbb {C})\times \mathrm {SL}_{2}(\mathbb {C})$. The latter has two non-compact real forms;
  - $\mathrm {SL}_{2}(\mathbb {R})\times \mathrm {SL}_{2}(\mathbb {R})$ and its corresponding orbit is $Y=S^{1}\times S^{1}$.
  - $\mathrm {SL}_{2}(\mathbb {C})$ embedded as an antiholomorphic diagonal and its orbit is given in Theorem 5.5 in [19]. One way to think of this real form is as the fixed point subgroup of the antiholomorphic involution defined by $\sigma (A,B)=(\bar{B},\bar{A})$. Then $\mathcal {M}_{1}=\{(z,\bar{z}):z\in \mathbb {P}_{1}\}$.
2. If $Z=\mathbb {P}_{2}$, then the complex Lie group of this surface is $\mathrm {SL}_{3}(\mathbb {C})$. The latter has two non-compact real forms, namely $\mathrm {SL}_{3}(\mathbb {R})$ and $\mathrm {SU}(1,2)$. The group $\mathrm {SL}_{3}(\mathbb {R})$ has only one orbit of dimension 2 in $\mathbb {P}_{2}$ which is isomorphic to the non-orientable projective plane $\mathbb {RP}_{2}$. Whereas the form $\mathrm {SU}(1,2)$ has three orbits in $\mathbb {P}_{2}$, two are open and diffeomorphic to the unit ball in $\mathbb {C}^{2}$ and the compact one is the boundary between the other two and has real dimension 3 (see the example in [19, Sect. 5.4.2]) and hence the latter real form does not take place in our setting. More generally, in a flag manifold, the only 2-dimensional homogeneous CR-submanifold of a simple Lie group is the real projective space $\mathbb {RP}_{2}$. See [19, Theorem 5.3].

$$\square $$

We note that the minimality condition is also not necessary here since *X* is simply-connected.

It remains to study the setting of a non-compact complex leaf-space *Z*. Huckleberry and Livorni in [22, Theorem, p. 1103] gave a complete list of homogeneous non-compact complex surfaces, and we state the list as follows:

##### Theorem 5.4

(Huckleberry and Livorni) *Let* *Z* *be a non-compact complex homogeneous surface of a non-solvable complex Lie group. Assume that the radical of this group does not act transitively on* *Z*. *If* *Z* *is not a holomorphically trivial* $\mathbb {C}^{*},$ *or* $\mathbb {C}$-*bundle over* $\mathbb {P}_{1}$, *then it is either*,

1. *a non-trivial* $\mathbb {C}^{*}$-*bundle over* $\mathbb {P}_{1},$
2. *a non-trivial positive line bundle over* $\mathbb {P}_{1},$
3. *the affine quadric* $Q_{[2]}:=\mathrm {SL}_{2}(\mathbb {C})/\mathbb {C}^{*},$ *or*
4. *the complement of a quadric curve* *C* *in* $\mathbb {P}_{2}$ *which is 2-to-1 covered by the previous case.*

*Moreover*, *in all cases the complex Lie group is* $\mathrm {SL}_{2}(\mathbb {C})$.

##### Proposition 5.4

(2-Dimensional non-compact leaf-spaces) *In codimension two if the complex leaf-space* *Z* *is not compact*, *then* *Z* *is either one of the following Stein surfaces*:

(I) *the affine quadric* $Q_{[2]}=\mathrm {SL}_{2}(\mathbb {C})/\mathbb {C}^{*}$. *In this case*, *the compact real leaf-space* *Y* *is orientable and isomorphic to* $S^{2}$.
(II) $\mathbb {P}_{2} \setminus C,$ *where* *C* *is a quadric curve in* $\mathbb {P}_{2}$. *In this case*, *the compact real leaf-space* *Y* *is non-orientable and isomorphic to* $\mathbb {RP}_{2}$.

##### Proof

Since the complex Lie group is semisimple, it follows that the leaf-space *Z* cannot be a holomorphically-trivial $\mathbb {C}$-bundle (resp. $\mathbb {C}^{*}$-bundle) over $\mathbb {P}_{1}$.

We now want to exclude the first two surfaces in Theorem 5.4 from our list;

$\bullet$ A non-trivial $\mathbb {C}^{*}$-bundle over $\mathbb {P}_{1}$; Clearly, this space is biholomorphic to $\mathrm {SL}_{2}(\mathbb {C})/ \widehat{H}$, where the connected component $\widehat{H}^{\circ}$ of the isotropy $\widehat{H}$ is isomorphic to $$\begin{pmatrix} 1&{}*\\ 0&{} 1 \end{pmatrix}$$. Since the action is algebraic then $\widehat{H}$ has finite connected components, i.e., $\widehat{H}=\mathbb {Z}_{n} \widehat{H}^{0}$, where the finite cyclic group $\mathbb {Z}_{n}$ refers to a diagonal subgroup of $\mathrm {SL}_{2}(\mathbb {C})$ isomorphic to the group of the nth roots of unity. Note that the induced orbit of the real form $\mathrm {SL}_{2}(\mathbb {R})/(\mathrm {SL}_{2}(\mathbb {R})\cap \widehat{H})\hookrightarrow \mathrm {SL}_{2}(\mathbb {C})/\widehat{H}$ is not compact since the orbits of the real unipotent subgroup $$\begin{pmatrix} 1&{}0\\ *&{}1 \end{pmatrix}\subset \mathrm {SL}_{2}(\mathbb {R})$$ are closed and diffeomorphic to $\mathbb {R}$. Thus, this situation does not occur. On the other hand, the induced orbit of the compact real form $\mathrm {SU}(2)$

$$\begin{aligned} \mathrm {SU}(2)/(\mathrm {SU}(2)\cap \widehat{H}) \hookrightarrow \mathrm {SL}_{2}(\mathbb {C})/\widehat{H} \end{aligned}$$

has dimension 3 because the isotropy $\mathrm {SU}(2)\cap H$ is finite, and thus this situation does not occur either.

$\bullet$ A positive line bundle over $\mathbb {P}_{1}$ requires a positive-dimensional radical (see [22, Lemma 1, p. 1103]) and our group is semisimple. Thus, this situation does not occur.

In contrast, the following cases can occur:

$\mathrm {(I)}$ *Z* is the affine quadric $\mathrm {SL}_{2}(\mathbb {C})/\mathbb {C}^{*}$, where $\mathbb {C}^{*}$ refers to the diagonal subgroup. Here *Y* is the orbit of the compact real form $\mathrm {SU}(2)$, i.e.,

$$\begin{aligned} Y= \mathrm {SU}(2)/(\mathrm {SU}(2)\cap \mathbb {C}^{*})= \mathrm {SU}(2)/\mathrm {SU}(1)\cong S^{2}. \end{aligned}$$

$\mathrm {(II)}$ *Z* is the complement of a quadric curve *C* in $\mathbb {P}_{2}$, i.e.,

$$\begin{aligned} \mathbb {P}_{2}\setminus \left\{ [X,Y,Z];\ X^{2}+Y^{2}+Z^{2}=0\right\} . \end{aligned}$$

It can be shown that the group $\mathrm {SL}_{2}(\mathbb {C})$ acts holomorphically on this surface and that the isotropy has two connected components and the connected component is isomorphic to the diagonal subgroup $\mathbb {C}^{*}$. Thus, $\mathbb {P}_{2}\setminus C=\mathrm {SL}_{2}(\mathbb {C})/\mathbb {Z}_{2}\mathbb {C}^{*}$. Therefore, we have the covering space

$$\begin{aligned} \mathrm {SL}_{2}(\mathbb {C})/\mathbb {C}^{*}\; \xrightarrow {\mathbb {Z}_{2}}\; \mathrm {SL}_{2}(\mathbb {C})/\mathbb {Z}_{2}\mathbb {C}^{*}. \end{aligned}$$

Note that the orbit of ${\text {SU}}(2)$ in the covering space is isomorphic to $S^{2}$ and hence the projection *Y* of this orbit is isomorphic to the non-orientable projective plane $\mathbb {RP}_{2}$. $\square$

The following table summarizes the above discussion, where $$\mathcal {M}_{1}$$ is as in Proposition 5.3, $$Q_{[2]}$$ is the 2-affine quadric, *C* is a quadric curve in $$\mathbb {P}_{2}$$, $$ B_{j}$$ is a Borel subgroup, and *P* is a parabolic subgroup.

**Table:** 

$\mathrm {codim}\; \varSigma$	*Z*	$Z=\widehat{S}/\widehat{H}$	*Y*	Real form of $\widehat{S}$
1	$\mathbb {P}_{1}$	$\mathrm {SL}_{2}(\mathbb {C})/B_{1}$	$S^{1}$	$\mathrm {SL}_{2}(\mathbb {R})$
2	$\mathbb {P}_{1}\times \mathbb {P}_{1}$	$\mathrm {SL}_{2}(\mathbb {C})\times \mathrm {SL}_{2}(\mathbb {C})\Big /B_{1}\times B_{2}$	$S^{1}\times S^{1}$	$\mathrm {SL}_{2}(\mathbb {R})\times \mathrm {SL}_{2}(\mathbb {R})$
2	$\mathbb {P}_{1}\times \mathbb {P}_{1}$	$\mathrm {SL}_{2}(\mathbb {C})\times \mathrm {SL}_{2}(\mathbb {C})\Big /B_{1}\times B_{2}$	$\mathcal {M}_{1}$	$\mathrm {SL}_{2}(\mathbb {C})$
2	$\mathbb {P}_{2}$	$\mathrm {SL}_{3}(\mathbb {C})/ P$	$\mathbb {RP}_{2}$	$\mathrm {SL}_{3}(\mathbb {R})$
2	$Q_{[2]}$	$\mathrm {SL}_{2}(\mathbb {C})/\mathbb {C}^{*}$	$S^{2}$	$\mathrm {SU}(2)$
2	$\mathbb {P}_{2}\setminus C$	$\mathrm {SL}_{2}(\mathbb {C})/\mathbb {Z}_{2}\mathbb {C}^{*}$	$\mathbb {RP}_{2}$	$\mathrm {SU}(2)$

Recall that $\mathrm {SL}_{2}(\mathbb {C})$ is the unique (up to isomorphism) complex semisimple Lie subgroup of $\mathrm {GL}_{2}(\mathbb {C})$. Therefore, if $\widehat{S}$ is a complex semisimple Lie group having irreducible 2-dimensional representation, then there exist two semisimple complex normal Lie subgroups $\widehat{S}_{1},\; \widehat{S}_{2}\lhd \widehat{S}$ such that $\widehat{S}$ decomposes as a locally direct product $\widehat{S}_{1} \cdot \widehat{S}_{2}$ where $\widehat{S}_{1}$ is acting trivially and $\widehat{S}_{2}$ is isomorphic to the usual action of $\mathrm {SL}_{2}(\mathbb {C})$ on $\mathbb {C}^{2}$. The following fact is well known, but we include it for completeness.

##### Lemma 5.6

*Let* $\widehat{S}$ *be a connected*, *semisimple*, *complex Lie group and* $\widehat{H}$ *be a parabolic subgroup of* $\widehat{S}$. *Suppose* $\widehat{J}$ *is any closed*, *complex subgroup of* $\widehat{S}$ *that contains* $\widehat{H}$. *Consider the induced homogeneous fibration* $\widehat{S}/\widehat{H} \rightarrow \widehat{S}/\widehat{J}$. *Then its fiber and base are flag manifolds. Moreover*, *if its base is* $\mathbb {P}_{1}$ *or a product of* $\mathbb {P}_{1}$’*s*, *then the bundle is holomorphically trivial.*

##### Proof

Since $\widehat{H}$ is parabolic in $\widehat{S}$, it contains a Borel subgroup of $\widehat{S}$. This Borel subgroup is then contained in $\widehat{J}$. Thus $\widehat{J}$ is parabolic and $\widehat{S}/\widehat{J}$ is a flag manifold. Moreover, the fiber is a compact homogeneous projective variety and hence is a flag manifold. Now, suppose that $\widehat{S}/\widehat{H}$ has the fibration $\widehat{S}/\widehat{H}\rightarrow \widehat{S}/\widehat{J}=\mathbb {P}_{1}$. Then as discussed above there exists two normal complex Lie subgroups $\widehat{S}_{1}$ and $\widehat{S}_{2}\cong \mathrm {SL}_{2}(\mathbb {C})$ of $\widehat{S}$ such that $\widehat{S}/\widehat{J}=\widehat{S}/\widehat{S}_{1}\widehat{H}$. Moreover, since the normal subgroup $\widehat{S}_{2}$ acts algebraically, then its orbits are closed and we have the fibration $\widehat{S}/\widehat{H} \xrightarrow {\widehat{S}_{2}/\widehat{S}_{2}\cap \widehat{H}} \widehat{S}/\widehat{S}_{2} \widehat{H}$. By the first paragraph of the lemma, the fiber $\widehat{S}_{2}/\widehat{S}_{2}\cap \widehat{H}$ is a flag manifold of dimension bigger than or equal to the dimension of $\widehat{S}/\widehat{S}_{1}\widehat{H}=\mathbb {P}_{1}$. But since $\mathbb {P}_{1}$ is the only flag manifold realized by the action of $\mathrm {SL}_{2}(\mathbb {C})$, we have $\widehat{S}_{2}/\widehat{S}_{2}\cap \widehat{H}= \widehat{S}/\widehat{S}_{1}\widehat{H}$, which implies that $\widehat{S}_{2}$-orbits form sections of the bundle $\widehat{S}/\widehat{H} \rightarrow \widehat{S}/\widehat{J}$, and for this reason this bundle is trivial. The same proof works for multiple copies of $\mathbb {P}_{1}$, since $\widehat{S}$ in this case splits off as the product $\widehat{S}_{1}\cdot \widehat{S}_{2}\cdots \widehat{S}_{n}$, where $\widehat{S}_{1}$ is acting trivially and $\widehat{S}_{j}$ is isomorphic to the usual action of ${\text {SL}}_{2}(\mathbb {C})$ on $\mathbb {C}^{2}$, for $j=2,\ldots , n$. $\square$

As a consequence, the following theorem specifies all possible total spaces of the bundle (8).

##### Theorem 5.5

*In codimension two.*

- *If* $X=\widehat{S}/\widehat{H}$ *is compact*, *then*
  1. $$X=\mathcal {L}\times \mathbb {P}_{1}\times \mathbb {P}_{1}$$ *and* $$\varSigma =\mathcal {L}\times S^{1}\times S^{1}$$.
  2. $$X=\mathcal {L}\times \mathbb {P}_{1}\times \mathbb {P}_{1}$$ *and* $$\varSigma =\mathcal {L}\times \mathcal {M}_{1}$$.
  3. *X* *is a* $\mathcal {L}$-*bundle over* $\mathbb {P}_{2}$ *and* $\varSigma$ *is a* $\mathcal {L}$-*bundle over* $\mathbb {RP}_{2}$.
- *If* $X=\widehat{S}/\widehat{H}$ *is not compact*, *then*
  1. *X* *is a* $\mathcal {L}$-*bundle over* $Q_{[2]}$ *and* $\varSigma$ *is a* $\mathcal {L}$-*bundle over* $S^{2}$.
  2. *X* *is a* $\mathcal {L}$-*bundle over* $\mathbb {P}_{2}\setminus C$ *and* $\varSigma$ *is a* $\mathcal {L}$-*bundle over* $\mathbb {RP}_{2}$.

On the other hand, when the leaf-space is the affine quadric $Q_{[2]}$, then the leaf-reduction is not necessarily trivial. In fact, the representation $\mathbb {C}^{*}\rightarrow \mathrm {Aut}(Q)$ implies that the isotropy $\mathbb {C}^{*}$ of the base does not need to act trivially on the fiber *Q*.

##### Remark 5.1

A totally real copy of $\mathbb {RP}_{2}$ in $\mathbb {P}_{2}$ can be embedded equivariantly in $\mathbb {P}_{2}\setminus C$ where *C* is a quadric curve in $\mathbb {P}_{2}$. To see this, let $\mathbb {RP}_{2}$ be an orbit of $\mathrm {SL}_{3}(\mathbb {R})$ as in Corollary 5.3. Since $\pi _{1}(\mathbb {RP}_{2})=\mathbb {Z}_{2}$, i.e., finite, then by Montgomery Theorem [27], the maximal compact subgroup $\mathrm {SO}(3,\mathbb {R})$ of $\mathrm {SL}_{3}(\mathbb {R})$ acts transitively. Thus, we have the following embedding in the orbit of the complex orthogonal subgroup

$$\begin{aligned} \mathbb {RP}_{2}= \mathrm {SO}(3,\mathbb {R})\cdot x_{0} \;\hookrightarrow \; Z:\;=\;\mathrm {SO}(3,\mathbb {C}) \cdot x_{0}\; \hookrightarrow \; \mathrm {SL}_{3}(\mathbb {C})\cdot x_{0}=\mathbb {P}_{2}. \end{aligned}$$

But since $\mathrm {SL}_{2}(\mathbb {C})$ (resp. $\mathrm {SU}(2)$) is the universal covering group of $\mathrm {SO}(3,\mathbb {C})$ (resp. $\mathrm {SO}(3,\mathbb {R})$), then one has the following orbits

$$\begin{aligned} \mathbb {RP}_{2}\; =\; \mathrm {SU}(2)/\mathbb {Z}_{2}\cdot S^{1}\; \hookrightarrow \; \mathrm {SL}_{2}(\mathbb {C})/\mathbb {Z}_{2}\cdot \mathbb {C}^{*}\; \cong \; \mathbb {P}_{2}\setminus C, \end{aligned}$$

where *C* is a quadric curve in $\mathbb {P}_{2}$.

### Summary

We summarize the classification results in this section in the following theorem.

#### Theorem 5.6

(Classification) *Suppose* $\varSigma :=G/H$ *is a compact, homogeneous, Leviflat CR-manifold of codimension one or two that is equivariantly embedded in a projective space* $\mathbb {P}_{n},$ *and let* $X:=\widehat{G}/\widehat{H}$ *be its globalization in* $\mathbb {P}_{n}$. *Then*, *the leaves of the Levi-foliation* $\mathscr {F}$ *are compact and hence biholomorphic to a flag manifold* *Q*. *Moreover*,

1. *if* $\mathrm {codim} \, \varSigma \; =\; 1,$ *then* $\varSigma \; =\; Q\; \times \; S^{1}\; \subset \; X\; =\; Q\;\times \; \mathbb {P}_{1}$ *or* $X\;=\;Q\;\times \; \mathbb {C}^{*},$
2. *if* $\mathrm {codim} \, \varSigma \;=\;2,$ *then either*
  - $$\varSigma \; =\; Q\; \times \; S^{1}\; \times \; S^{1}\;\subset \; X\; =\; Q \; \times \; \mathbb {P}_{1}\; \times \; \mathbb {P}_{1}$$ (*resp.* $$X\; =\; Q\; \times \; \mathbb {P}_{1}\;\times \; \mathbb {C}^{*}$$ or $$X\; =\; Q\; \times \; \mathbb {C}^{*}\; \times \;\mathbb {C}^{*}).$$
  - $$X=Q\times \mathbb {P}_{1}\times \mathbb {P}_{1}$$ *and* $$\varSigma =Q\times \mathcal {M}_{1}$$.
  - $\varSigma$ *is a* *Q*-*bundle over* $S^{2}$ *and* *X* *is a* *Q*-*bundle over the* 2-*dimensional affine quadric* $Q_{[2]}$.
  - $\varSigma$ *is a* *Q*-*bundle over* $\mathbb {RP}_{2}$ *and* *X* *is a* *Q*-*bundle over* $\mathbb {P}_{2}\setminus C$ *where* *C* *is a quadric curve in* $\mathbb {P}_{2}$. (*This situation will occur only when* $\varSigma$ *is non-orientable. It is* 2 *to* 1 *covered by the previous case.*)

## General case

### Statement of the main theorem

#### Theorem 6.1

(Main Theorem) *Let* *G* *be a connected and simply-connected Lie group*, *and* $\varSigma :=G/H$ *be a compact*, *generic*, *homogeneous*, *Leviflat CR-manifold of codimension one or two. Consider the CR-normalizer fibration* (*see Theorem* 2.2),

$$\begin{aligned} \varSigma \;=\; G/ H \longrightarrow G/J\; =:\; M \;\hookrightarrow \; \mathbb {P}_{n}. \end{aligned}$$

*Then*, $\varSigma$ *is a finite covering space of* $\tilde{\varSigma }:=G/\tilde{H}$ *with the following properties:*

$\bullet$ *The fiber* *F* *of the following fibration*

$$\begin{aligned} \tilde{\varSigma }\;=\; G/\tilde{H} \xrightarrow { F\;:=\; J/\tilde{H} } G/J\; =\; M \end{aligned}$$

*is a connected*, *compact*, *parallelizable*, *Leviflat* , *homogeneous CR-submanifold of* $\tilde{\varSigma },$ *and the base* *M* *is a compact*, *projective Leviflat CR-manifold. Moreover* (*see Lemma*2.1),

$$\begin{aligned} \mathrm {codim}\; F \; +\; \mathrm {codim}\; M \; =\; \mathrm {codim}\;\tilde{\varSigma }. \end{aligned}$$

$\bullet$ *Let* $\widehat{G}$ *be the connected and simply-connected complex Lie group corresponding to the complexified Lie algebra* $\widehat{\mathfrak {g}}:=\mathfrak {g}+i\mathfrak {g}$, *where* $\mathfrak {g}$ *is the Lie algebra of* *G*. *Then* $\tilde{\varSigma }$ *possesses a* $\widehat{G}$-*globalization*, *i.e.*, *a complex homogeneous manifold* $X:=\widehat{G}/\widehat{H}$ *with* *G* *is a Lie subgroup of* $\widehat{G}$ *and* $\widehat{H}\cap G=\tilde{H}$. *In particular*, *the following diagram of fiber bundles exists*,$\bullet$ *The spaces* $F,\; \widehat{F},\; M$ , *and* *Y* *in the above diagram are described as follows*, (*in the following*, *Q*,  $Q_{[2]}$ , *and* *C* *stand for a flag manifold*, *the* 2-*dimensional affine quadric*, *and a quadric curve in* $\mathbb {P}_{2},$ *respectively. Also see Table* 1, *for the CT-notation*)

(I) *In codimension one*, *and when*

- *the Levi-foliation is dense, then* $Y=M=Q,$ $\widehat{F}=\mathrm {CT}_{1},$ $F=\mathrm {ct}_{1}$.
- *the Levi-foliation is compact*, *then either*
  1. $$Y=M=Q,$$ $$\widehat{F}=\mathrm {CT}^{*}_{0},$$ $$F=\mathrm {ct}^{*}_{0}$$.
  2. $$Y=Q\times \mathbb {P}_{1}$$ (resp. $$Y=Q\times \mathbb {C}^{*}$$), $$M=Q\times S^{1},$$ $$\widehat{F}=F=\mathrm {CT}_{0}$$.

(II) *In codimension two*, *and when*

- *the Levi-foliation is dense*, *then either*
  1. $$Y=M=Q,$$ $$\widehat{F}=\mathrm {CT}_{2},$$ $$F=\mathrm {ct}_{2}$$.
  2. $$Y=M=Q,$$ $$\widehat{F}=\mathrm {CT}_{1,1},$$ $$F=\mathrm {ct}_{1,1}$$.
- *the Levi-foliation is compact*, *then either*
  1. $$Y=M=Q,$$ $$\widehat{F}=\mathrm {CT}^{**}_{0},$$ $$F=\mathrm {ct}^{**}_{0}$$.
  2. $$Y=Q\times \mathbb {P}_{1}$$ (*resp.* $$Y=Q\times \mathbb {C}^{*}$$), $$M=Q\times S^{1},$$ $$\widehat{F}=\mathrm {CT}^{*}_{0},$$ $$F=\mathrm {ct}^{*}_{0}$$.
  3. $$Y=Q\times \mathbb {P}_{1}\times \mathbb {P}_{1}$$ (*resp.* $$Y=Q\times \mathbb {P}_{1}\times \mathbb {C}^{*},$$ or $$Y=Q\times \mathbb {C}^{*}\times \mathbb {C}^{*}$$),    $$M=Q\times S^{1}\times S^{1},$$    $$\widehat{F}=F=\mathrm {CT}_{0}$$.
  4. $Y=Q\times \mathbb {P}_{1}\times \mathbb {P}_{1}$ *and* $M=Q\times \mathcal {M}_{1}$ (*see Proposition* 5.3).
  5. *Y* *is a* *Q*-*bundle over* $Q_{[2]}$, *M* *is a* *Q*-*bundle over* $S^{2},$ $\widehat{F}=F=\mathrm {CT}_{0}$.
  6. *Y* *is a* *Q*-*bundle over* $\mathbb {P}_{2}\setminus C,$ *M* *is a* *Q*-*bundle over* $\mathbb {RP}_{2},$ (*Y* *in this case is* 2-*to*-1 *covered by the previous case*) $\widehat{F}=F=\mathrm {CT}_{0}$.
- *the Levi-foliation is neither compact nor dense*, *then either*
  1. $$Y=M=Q,$$ $$\widehat{F}=\mathrm {CT}^{*}_{1},$$ $$F=\mathrm {ct}^{*}_{1}$$.
  2. $$Y=Q\times \mathbb {P}_{1}$$ (*resp.* $$Y=Q\times \mathbb {C}^{*}$$), $$M=Q\times S^{1},$$ $$\widehat{F}=\mathrm {CT}_{1},$$ $$F=\mathrm {ct}_{1}$$.

## The globalization

Given a homogeneous CR-manifold $\varSigma = G/H$, if there exists a (minimal) complex Lie group $\widehat{G}$ containing *G* as a subgroup along with a closed complex subgroup $\widehat{H}$ such that the *G*-orbit in $\widehat{G}/\widehat{H}$ is CR-isomorphic to $\varSigma$, then we call $\widehat{G}/\widehat{H}$ a globalization of $\varSigma$. Such a globalization need not exist, e.g., see [6, Sect. 2, Example]. However, we prove in this section that every compact, Leviflat $\varSigma$ of codimension two or less is globalizable.

First we consider what happens when the base *Z* of the CR-normalizer fibration $G/H \rightarrow G/N = Z$ is a flag manifold. The case of a general homogeneous CR-hypersurface with *Z* a flag manifold is discussed in [24] (Theorem 2) and was handled in detail in Richthofer’s thesis [35] (Satz 1 in Sect. 1:4:3). The result is simply that such actions are uniquely globalizable. Using our special Leviflat setting, we give a simple proof of this fact here.

### Proposition 7.1

[35, Sect. 1.4.3, Satz 1] *Assume that the base of the CR- normalizer fibration of the compact Leviflat homogeneous* $\varSigma$ *is a flag manifold and* $\varSigma$ *has codimension less than or equal to two. Then* $\varSigma$ *admits a globalization.*

### Proof

Since *Z* is a compact complex manifold, it is immediate that $G_{\mathcal {L}}$ acts transitively on it and thus it is a homogeneous space $Z = \widehat{S}/\widehat{Q}$ of a product $\widehat{S}$ of simple factors of $G_{\mathcal {L}}$. It is enough to globalize the *G*-action on $G/H^{\circ}$ and for that we consider the fiber $P := N/H^{\circ}$ of $G/H^{\circ} \rightarrow G/N$. This is a *P*-principal bundle $\mathcal {P}$ over $Z = \widehat{S}/\widehat{Q}$. Let $\widehat{U}$ be a local section (contained in $\widehat{S}$) of the fibration $\widehat{S} \rightarrow \widehat{S}/\widehat{Q}$. Locally, the CR-manifold $\varSigma$ is just the CR-product $\widehat{U}\times P$. More explicitly, if $\widehat{P}$ is a globalization of *P* (see remark below for its existence), then the embedding $\iota : \widehat{U} \times P \hookrightarrow \widehat{U} \times \widehat{P}$ defines the sheaf of CR-functions on $\widehat{U} \times P$ by the isomorphism $\iota : \mathcal {O}_{\widehat{U} \times \widehat{P}}{\hookrightarrow }^{*} \mathcal {O}^{CR}_{\widehat{U}\times P}$. We have conveniently chosen $\widehat{Q}$ to be in $G_{\mathcal {L}}$ whose action on $\widehat{P}$ is an extension of its action on P. Thus, we have the embedding

$$\begin{aligned} \widehat{S} \times _{\widehat{Q}} P \hookrightarrow \widehat{S} \times _{\widehat{Q}} \widehat{P} . \end{aligned}$$

Locally, over $\widehat{U}$ these twisted products are quotients of $\widehat{U}\widehat{Q}\times P$ and $\widehat{U}\widehat{Q}\times \widehat{P}$ by the diagonal $\widehat{Q}$-action (on the right on the first factor and on the left on the second). Since $\widehat{U} \times P$ and $\widehat{U} \times \widehat{P}$ are sections of these fibrations, it follows that the CR-functions on the former are just the pullbacks of the holomorphic functions on the latter. Thus, the above embedding realizes $\varSigma = \widehat{S} \times _{\widehat{Q}} P$ as a CR-submanifold of the complex manifold $\widehat{S} \times _{\widehat{Q}} \widehat{P}$.

Now, ineffectively, *J* of the *G*-action on the base *G*/*N* acts (on the left) on *P* and globalizes to a left-action of $\widehat{J}$ on $\widehat{P}$. Since the left-action of $\widehat{S}$ on $\widehat{S} \times _{\widehat{Q}}\widehat{P}$ is already globalized, it follows that the *G*-action on $\varSigma$ is globalized to a $\widehat{G}$-action on this complex principal bundle space over *Z*. $\square$

Next we are going to prove the existence of the globalization when the fiber of the CR-normalizer fibration is complex. In order to do this suppose $\varSigma =G/H$ is a compact homogeneous CR-manifold, where *G* is a connected and simply-connected Lie group. Let

$$\begin{aligned} \varSigma \;=\; G/ H \longrightarrow G/J\; =:\; M \;\hookrightarrow \; \mathbb {P}_{n} \end{aligned}$$

be the CR-normalizer fibration. We can consider the manifold $\tilde{\varSigma }$ such that

$$\begin{aligned} \varSigma \;=\;G/H\xrightarrow {\mathbb {Z}_{p}} \tilde{\varSigma }\;=\; G/\tilde{H} \xrightarrow { F } M\;=\; G/J\hookrightarrow \mathbb {P}_{n} \end{aligned}$$

has a *connected* parallelizable fiber $F:=J/\tilde{H}=:L/\varGamma$, where *L* is a connected Lie group and $\varGamma$ is a discrete subgroup of *L*. Note that since *F* is assumed to be connected, the connected component $J^{0}$ of *J* acts transitively on *F*. Also in codimensions one and two, *F* possesses a connected parallelizable globalization $\widehat{F}:=\widehat{L}/\varGamma$ (see Sect. 4).

### Remark 7.1

Consider the globalization $\widehat{G}/\widehat{J}$ of the base $G/J\hookrightarrow \mathbb {P}_{n}$ in the fibration above. Note that $\widehat{J}$ acts transitively and holomorphically on the connected complex manifold $\widehat{F}$ if and only if its connected component $\widehat{J}^{0}$ does so. Thus, if $\widehat{F}$ possesses a $\widehat{J}^{0}$-globalization, then we are naturally led to consider the $\widehat{G}$-globalization of $\tilde{\varSigma }$ given by the construction

$$\begin{aligned} X\; := \; \widehat{G}\times _{\widehat{J}^{0}}\widehat{F}\; =\; \widehat{G}\times _{\widehat{J}}\widehat{F}. \end{aligned}$$

The following lemma works for any codimension.

### Lemma 7.1

*If the fiber* *F* *is complex*, *i.e.*, $F=\widehat{F}$ *then* $\widehat{J}$ *acts on* $\widehat{F}$ *holomorphically and transitively.*

### Proof

If $G_{\mathcal {L}}$ is the leaf-stabilizer of the leaf $\mathcal {L}$ through the base point in $\varSigma$ then the complex Lie subgroup $\widehat{ G_{\mathcal {L}}}$ of $\widehat{G}$ corresponding to the complexified Lie algebra $\widehat{\mathfrak {g}_{\mathcal {L}}}:= \mathfrak {g}_{\mathcal {L}}+i\mathfrak {g}_{\mathcal {L}}$ also stabilizes $\mathcal {L}$ in *X*. Therefore, if $F=\widehat{F}$ is complex, then it is contained in the leaf $\mathcal {L}$. Thus $\widehat{J}<\widehat{G_{\mathcal {L}}}$ is the complex stabilizer of $\widehat{F}$ in *X*, i.e., $\widehat{J}$ acts transitively and holomorphically on $\widehat{F}$. $\square$

Now the (local) holomorphic action of the Lie algebra $\widehat{\mathfrak {j}}$ of $\widehat{J}^{0}$ on $\widehat{F}$ induces a local holomorphic action of $\widehat{J}^{0}$. Thus, since in codimension one or two, the fiber *F* is globalizable, then the *universal covering* $\widehat{J}_{1}$ of $\widehat{J}^{0}$ acts transitively and holomorphically on its globalization $\widehat{F}$. The question now arises whether the $\widehat{J}_{1}$-action on $\widehat{F}$ descends to a $\widehat{J}^{0}$-action.

To answer this question, we follow the proof given in [19, Sect. 3.1] after restricting ourselves to the case where the fiber *F* is connected.

### Proposition 7.2

*Suppose that the inclusion map* $J^{0}\hookrightarrow \widehat{J}^{0}$ *induces a surjective homomorphism of the fundamental groups* $\pi _{1}(J^{0})\rightarrow \pi _{1}(\widehat{J}^{0})$, *then the* $\widehat{J}^{0}_{1}$-*action on* $\widehat{F}$ *descends to a* $\widehat{J}^{0}$-*action.*

### Proof

Let $J_{1}$ be the lift of the $J^{0}$ into the universal covering $\widehat{J}_{1}$ of $\widehat{J}^{0}$. Let $\varLambda$ be the kernel of the covering $\widehat{J}_{1}\rightarrow \widehat{J}^{0}$. The surjectivity of the homomorphism $\pi _{1}(J^{0})\rightarrow \pi _{1}(\widehat{J}^{0})$ implies that the kernel of the covering $J_{1}\rightarrow J^{0}$ is also $\varLambda$, which in turn means that $\varLambda$ acts trivially on the fiber $\widehat{F}$ since it acts trivially on *F*. Thus, the action of $\widehat{J}_{1}$ descends to an action of $\widehat{J}_{1}/\varLambda = \widehat{J}^{0}$. $\square$

In practice, we are only able to answer such a homotopy question modulo the ineffectivity of the action on the base of the bundle. We now fix the following notation; Let $\widehat{I}$ be ineffectively of the $\widehat{G}$-action on $\widehat{G}/\widehat{J}$, and $\widehat{I}^{0}$ be its connected component. Also let $I^{0}$ be the connected component of the *G*-ineffectively $I:= \widehat{I}\cap G$.

**Condition (C)** The inclusion of Lie subgroups,

$$\begin{aligned} J^{0}/(J^{0}\cap \widehat{I}^{0})\; \hookrightarrow \; \widehat{J}^{0}/\widehat{I}^{0} \end{aligned}$$

induces a surjective homomorphism of the fundamental groups,

$$\begin{aligned} \pi _{1}\left( J^{0}/J^{0}\cap \widehat{I}^{0}\right) \;\longrightarrow \; \pi _{1}(\widehat{J}^{0}/\widehat{I}^{0}). \end{aligned}$$

### Proposition 7.3

*Suppose that*

9 $$\begin{aligned} J^{0}\cap \widehat{I}^{0} \; = \;I^{0}. \end{aligned}$$

*If Condition* (*C*) *is fulfilled*, *then the* $\widehat{J}_{1}$ *-action on* $\widehat{F}$ *descends to a* $\widehat{J}^{0}$ *-action.*

### Proof

Consider the homotopy sequences of the principal bundles:

$$\begin{aligned} \widehat{I}^{0}\hookrightarrow \widehat{J}^{0}\rightarrow \widehat{J}^{0}/\widehat{I}^{0} \quad \text {and} \quad I^{0} \hookrightarrow J^{0}\rightarrow J^{0}/I^{0}. \end{aligned}$$

Since $\widehat{I}^{0}$ (resp. $I^{0}$) is a normal Lie subgroup of a simply-connected Lie group $\widehat{G}$ (resp. *G*), then $\pi _{1}(\widehat{I}^{0})=\pi _{1}(I^{0})=0$. Thus,

$$\begin{aligned} \pi _{1}(J^{0}) \;&=\; \pi _{1}(J^{0}/I^{0}),\\ \pi _{1}(\widehat{J}^{0}) \;&=\; \pi _{1}(\widehat{J}^{0}/\widehat{I}^{0}). \end{aligned}$$

By Condition (C), we have $\pi _{1}( J^{0}) \rightarrow \pi _{1}(\widehat{J}^{0})$ is surjective and the proof follows from Proposition 7.2. $\square$

The following Lemma (see Lemma 3.1 in [19]) gives a sufficient condition for (9) in Proposition 7.3 to take place.

### Lemma 7.2

*Let* $\widehat{N}$ *be a connected complex normal subgroup of* $\widehat{G}$ *that contains the commutator* $\widehat{R}'$ *of the radical* $\widehat{R}$. *Then* $G\cap \widehat{N}$ *is connected.*

In our setting, Theorem 5.1 implies that $\widehat{R}$ always acts as an Abelian group on the base $\widehat{G}/\widehat{J}$ of the CR-normalizer fibration, i.e., $\widehat{R}'\subset \widehat{I}$. As a consequence, $G\cap \widehat{I}^{0}$ is connected, and therefore $J^{0}\cap \widehat{I}^{0}=I^{0}$. Thus, by Proposition 7.3, one has the following corollary.

### Corollary 7.1

*If the inclusion* $J^{0}/I^{0} \hookrightarrow \widehat{J}^{0}/\widehat{I}^{0}$ *induces a surjective homomorphism of the fundamental groups*, *then the* $\widehat{J}_{1}$-*action on* $\widehat{F}$ *descends to a* $\widehat{J}$-*action.*

Finally, we consider the general case where the codimension is less than or equal to two.

### Theorem 7.1

(Existence of Globalization) *Suppose* $\varSigma = G/H$ *is a compact*, *Leviflat*, *homogeneous CR-manifold having codimension less than or equal to two. Then* $\varSigma$ *possesses a* $\widehat{G}$-*globalization.*

### Proof

Note that $\widehat{J}^{0}/\widehat{I}^{0}$ is the connected component of the isotropy of the transitive $(\widehat{S}/\widehat{S}\cap \widehat{I}^{0})$-action on $\widehat{S}/\widehat{S}\cap \widehat{J}$. From the list in Theorem 5.6, when $F\not =\widehat{F}$, one can see that $\widehat{J}^{0}/\widehat{I}^{0}$ and $J^{0}/I^{0}$ are homotopic equivalent. By Corollary 7.1, the result follows. $\square$

## References

[1] Abe Y, Kopfermann K (2001). Toroidal Groups: Line Bundles, Cohomology and Quasi-Abelian Varieties. Lecture Notes in Mathematics.

[2] Al-Abdallah, A.R.: Compact homogeneous Leviflat CR-manifolds. PhD Dissertation, University of Regina (2020)

[3] Andreotti A, Fredricks GA (1979). Embeddability of real analytic Cauchy–Riemann manifolds. Ann. Sc. Norm. Super. Pisa.

[4] Akhiezer D (1984). Invariant analytic hypersurfaces in complex nilpotent Lie groups. Ann. Glob. Anal. Geom..

[5] Auslander L (1963). On radicals of discrete subgroups of Lie groups. Am. J. Math..

[6] Azad H, Huckleberry A, Richthofer W (1985). Homogeneous CR-manifolds. J. Reine Angew. Math..

[7] Barth W, Otte M (1969). Üper fast-uniforme Untergruppen komplexer Liegruppen und auflösbare komplexe Mannigfaltigkeiten. Comment. Math. Helv..

[8] Boggess A (1991). CR Manifolds and the Tangential Cauchy–Riemann Complex, Studies in Advanced Mathematics.

[9] Borel A (1985). On affine algebraic homogeneous spaces. Arch. Math..

[10] Borel A (1991). Linear Algebraic Groups. Graduate Texts in Mathematics.

[11] Borel A, Remmert R (1962). Über kompakte homogene Kählersche Mannigfaltigkeiten. Math. Ann..

[12] Borel A, Serre J (1950). Impossibilité de fibrer un espace euclidien par des fibres compactes. C. R. Acad. Sci. Paris.

[13] Chevalley C (1941). On the topological structure of solvable groups. Ann. Math..

[14] Chevalley C (1951). Théorie des groupes de Lie. II, Groupes Algébriques, Act. Sci. Ind., no. 1151.

[15] Chirka EM (1991). Introduction to the geometry of CR-manifolds. Russ. Math. Surv..

[16] Fulton W, Harris J (2004). Representation Theory: A First Course. Graduate Texts in Mathematics.

[17] Freeman M (1974). Local complex foliation of real submanifolds. Math. Ann..

[18] Gilligan B, Huckleberry AT (1978). On non-compact complex nil-manifolds. Math. Ann..

[19] Gilligan B, Huckleberry AT (2009). Fibrations and globalizations of compact homogeneous CR-manifolds. Izv. Math..

[20] Gilligan B (1981). Ends of complex homogeneous manifolds having non-constant holomorphic functions. Arch. Math..

[21] Gilligan B (2017). Levi’s problem for pseudoconvex homogeneous manifolds. Can. Math. Bull..

[22] Huckleberry AT, Livorni EL (1981). A classification of homogeneous surfaces. Can. J. Math..

[23] Huckleberry, A.T., Oeljeklaus, E.: Homogeneous spaces from a complex analytic view-point. In: Manifolds and Lie Groups (Papers in Honor of Y. Matsushima). Progress in Mathematics. Birkhäuser, Boston (1981)

[24] Huckleberry, A.T., Richthofer, W.: Recent developments in homogeneous CR-hypersurfaces. In: Contributions to Several Complex Variables, Aspects in Mathematics, vol E9, pp. 149–177. Vieweg, Braunschweig (1986)

[25] Humphreys JE (1975). Linear Algebraic Groups, Graduate Texts in Mathematics.

[26] Jacobowitz H (1990). An Introduction to CR Structures, Mathematical Surveys and Monographs 32.

[27] Montgomery D (1950). Simply connected homogeneous spaces. Proc. Am. Math. Soc..

[28] Mostow GD (1950). The extensibility of local Lie groups of transformations and groups on surfaces. Ann. Math..

[29] Mostow GD (1954). Factor spaces of solvable groups. Ann. Math..

[30] Mostow GD (1971). Some applications of representative functions to solvmanifolds. Am. J. Math..

[31] Oeljeklaus, E.: Hyperflächen und Geradenbündel auf homogenen komplexen Mannigfaltigkeiten, Schriftenreihe des Mathematischen Instituts der Universität Münster, Ser. 2, Heft 36, Münster (1985)

[32] Pinchuk S (1992). CR transformations of real manifolds in $$\mathbb{C}^{n}$$. Indiana Univ. Math. J..

[33] Rea C (1972). Levi-flat submanifolds and holomorphic extension of foliations. Ann. Sc. Norm. Super. Pisa.

[34] Reeb G (1952). Sur certaines propriétés topologiques des variétés feuilletées, Actual Sci. Ind., No. 1183.

[35] Richthofer, W.: Homogene CR-Mannigfaltigkeiten. PhD Dissertation, Ruhr-Universität Bochum (1985)

[36] Sommer F (1958). Komplex-analytische Blätterung reeller Mannigfaltigkeiten im $${\mathbb{C}}^{n}$$. Math. Ann..

[37] Springer TA (1998). Linear Algebraic Groups, Progress in Mathematics, 2nd edn.

[38] Sullivan D (1976). A counterexample to the periodic orbit conjecture. Publ. Math. IHES.

[39] Tits J (1962). Espaces homogènes complexes compacts. Comment. Math. Helv..

[40] Vogt C (1982). Line bundles on toroidal groups. J. Reine Angew. Math..

